# 1,4-Dihydropyrrolo[3,2-b]Pyrroles Containing New A-D-A System: Synthesis and Investigation of Their Photophysical Properties

**DOI:** 10.1007/s10895-025-04240-y

**Published:** 2025-03-21

**Authors:** Guler Yagiz Erdemir

**Affiliations:** Department of Chemistry, Faculty of Science, Gazi University, Ankara, 06560 Turkey

**Keywords:** TAPP, Acceptor-donor-acceptor Group, Stokes shift, Vilsmeier-haack

## Abstract

Herein, new tetraarylpyrrolo[3,2-b]pyrroles (TAPP) containing different substituents (**4a-c**,** 5a-c**) on the *N*-phenyl rings were synthesized in high yields using a one-pot method. The 3,6 positions of the pyrrolopyrol ring of the **4a-c** compound were formylated by Vilsmeier-Haack reaction to synthesize a new pyrrolopyrol compound containing the acceptor-donor-acceptor (A-D-A) system (**5a-c**). The photophysical properties of all obtained derivatives were investigated in different solvents. According to the results obtained, the absorptions of derivatives **4a-c** were recorded around 400 nm and their emission intensities appeared around 450 nm. The absorption intensities of derivatives **4b** and **5b** were recorded as 400 nm and 350 nm, respectively, while their emission intensities were observed as 455 and 440. In the A-D-A system formed as a result of the bonding of electron-withdrawing formyl groups at position 3,6 of the TAPP structure, a high Stokes shift occurred, while 50 nm hypsochromic shifts were observed in its absorption.

**Supplementary Information:**

The online version contains supplementary material available at 10.1007/s10895-025-04240-y.

## Introduction

Organic materials are structures preferred in a wide range of fields, from textile chemistry to medical chemistry and environmental chemistry to the design of optoelectronic materials due to their low mass and high efficiency [1–6]. With the increasing technology day by day, the need for newly designed molecules to develop many applications such as organic solar cells (OSCs), organic light-emitting diodes (OLEDs), and bioimaging is increasing [7–11]. It is very important to create optoelectronic systems with new design molecules, high bathochromic shifts and extended Stokes shifts and strong charge transfer (CT) that can be more effective in these technological applications [9, 12–13]. In applications such as OLED and OFET, the formation of structures with low molecular weight and high quantum efficiency is of great importance, and studies in this direction are gaining momentum every day and maintaining their place in the literature [7–10]. However, the low dye capacities and efficiency problems of D-A type systems, which are increasing day by day, cast a shadow on the application areas of these studies. Dye with enhanced optoelectronic capabilities can be designed by varying the strength of electron-donating and/or electron-withdrawing groups, as is well known [12–14].

Many electron donor groups, such as alkyl/arylamine and methoxy-substituted aromatic rings, have been described in the literature [15–16]. Structures including these groups are frequently preferred in pull-push systems due to their high quantum yields and Stokes shifts. 1,4-Dihydropyrrolo[3,2-b]pyrroles (DHPPs) have attracted attention, especially in recent years, with their strong electron supply, and their photophysical properties have been improved day by day with their extended π-conjugated systems in 2,5 positions [9, 17–21]. These cyclic structures have brought about great developments in their synthesis thanks to the one-pot method developed by Gryko and his group [17]. Remarkably, they are effective structures in pull push systems, OLED, OFET, and 2PA applications due to the red shift in emission due to phenyl rings in 1,4 and 2,5 positions, extended π-conjugated systems, and high quantum yields. In the literature, it is commonly seen that TAPP derivatives are derived from 2,5-positions because these positions contribute directly to π-conjugation and help to optimize the planarity and electronic properties of the molecule. However, the importance of the 1.4 position has not been clearly demonstrated in the literature, which has led to a limited number of studies on these positions. However, these locations are very important because of these positions are directly connected to pyrrole nitrogens and allow different modifications in terms of electron density and steric interactions. Functionalization at 1,4-positions allows to regulate intramolecular charge transfer properties by affecting the conjugation system of the molecule more locally, fine-tuning the optoelectronic performance and optimizing properties such as aggregation-induced emission (AIE) or mechanochromic fluorescence (MFC) [22]. Moreover, functionalization of 3,6-positions in TAPP derivatives is an important strategy to fine-tune the electronic structure of the molecule, optimize the conjugation effect, and direct the optoelectronic properties. Although these positions are not directly linked to the main conjugation pathway, they can affect the electronic distribution, allowing for precise regulation of the HOMO-LUMO energy levels. In particular, the addition of sterically bulky groups to these positions can reduce aggregation-induced quenching (ACQ) by blocking intermolecular π-π interactions and maintain bright fluorescent properties. In this respect, derivatization from 3,6-positions may offer researchers an alternative and powerful approach in the design of next-generation optoelectronic materials [23–25]. In this context, our hypothesis in this study is to form A-D-A type polyfunctional formyl-PP-formyl structures by forming formyl groups, which have high functional group properties and are also strong electron-withdrawing, at the 3,6 positions of DHPPs, which are strong electron donors [12–14, 17, 20, 21]. For this purpose, our first step is to synthesize a series of TAPP structures, especially those with different phenyl substituted in 1,4-positions, and to discuss their photophysical properties. Afterward, the aim was to synthesize formylated-TAPP structures by Vilsmeier–Haack reaction due to the high electron density at the 3,6 positions of these TAPP structures. After the structural characterizations of all obtained compounds were completed, their photophysical properties were examined, and quantum yields were calculated.

## Experimental

### Materials

All chemicals were bought from BLD company. The solvents in the synthesized step were purchased of analytical grade but the others solvent for purification steps purified in these steps using distillation method. A Perkin Elmer spectrophotometer was used to record Fourier transform infrared (FTIR) spectra on ATR. The ^1^H and ^13^C NMR spectra were obtained using the Bruker Avanced DPX 300 Spectrometer at 500 and 126 MHz, respectively. The coupling constant values were given in Hertz. S (singlet), d (doublet), t (triplet), q (quartet), m (multiplet), and so on were the mentioned splitting patterns. In relation to the internal standard tetramethylsilane (δ = 0.00 ppm), chemical changes were measured in parts per million. HRMS data was obtained using the Agilent LC/MS-High Resolution Quadrupole Mass Time-of-Flight (Q-TOF) instrument. The purity of analytical materials was confirmed and reaction progress was routinely tracked using thin layer chromatography (TLC). UV light was used for the detection of spots. UV-vis absorption and fluorescence spectra were screened with the HORIBA Duetta fluorescence and absorption spectrophotometer.

### General Synthesis of TAPPs (4a-c)

**Scheme 1:**
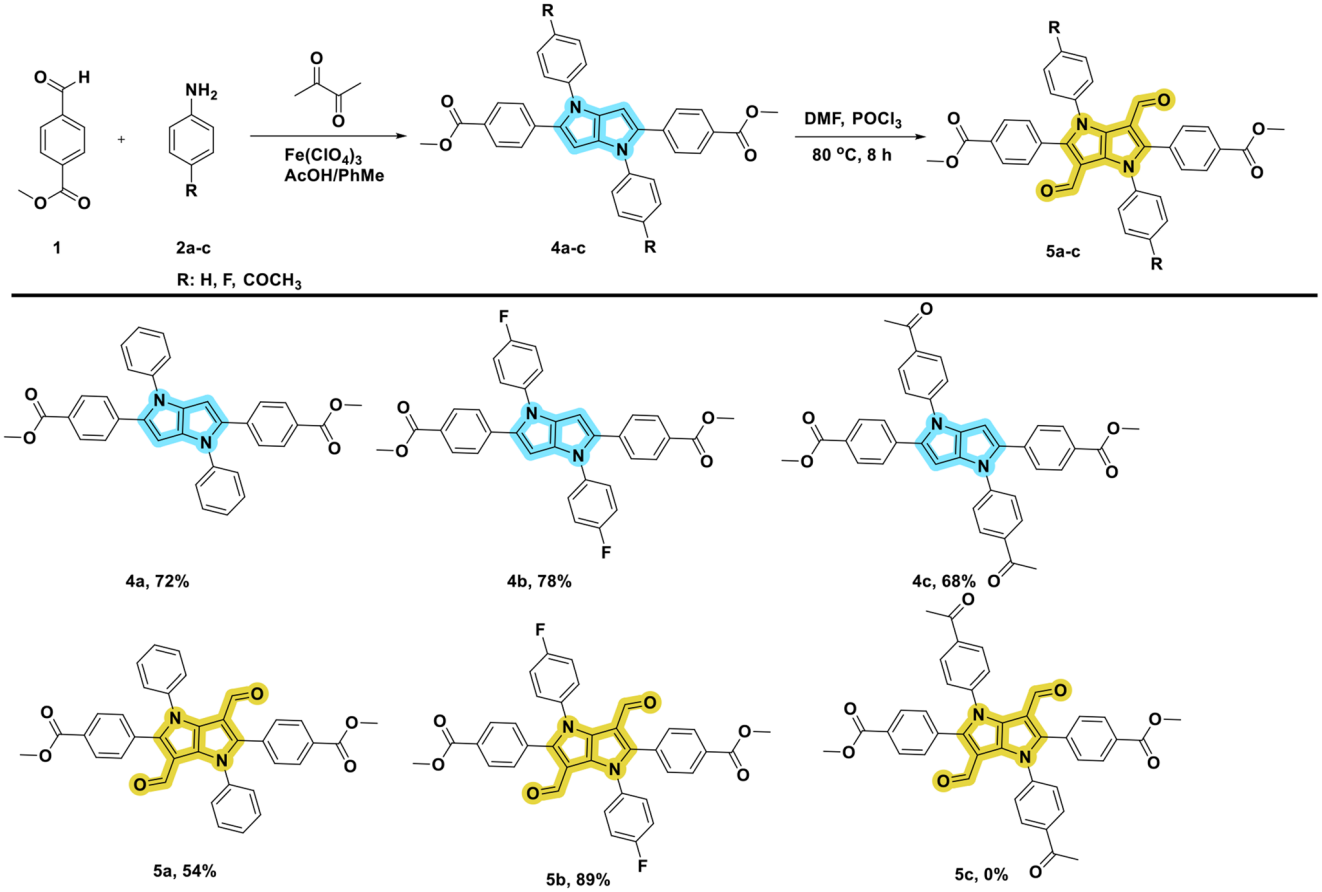
Synthesis of the target molecules

Aniline (2 mmol), aldehyde (2 mmol), and solvent system (acetic acid/toluene: 2/2 mL) were added to a round-bottomed and wide-mouthed reaction vessel and stirred for 2 h at 50 ℃. Then, iron (III) perchlorate (3 mmol%) and diacetyl (1 mmol) were added to this mixture and stirred at the same temperature overnight. After this period, 5 mL of ACN was added to the mixture, heated and vibrated, and hot filtration was performed. The solids obtained were washed twice with ACN, and pure TAPP structures were obtained [9, 26] (Scheme 1).

Dimethyl 4,4’-(1,4-diphenyl-1,4-dihydropyrrolo[3,2-b]pyrrole-2,5-diyl)dibenzoate (4a): Yellow solid, 78%, mp.: 276 ^o^C. IR spectrum, ν, cm^–1^: 3105, 2949, 1712, 1598. ^1^H NMR (500 MHz, CDCl_3_) δ 7.89 (d, *J =* 8.2 Hz, 4 H), 7.88 7.39 (t, *J* = 7.6 Hz, 4 H), 7.87–7.26 (m, 10 H), 6.51 (s, 2 H), 3.88 (s, 6 H). ^13^C NMR (126 MHz, CDCl_3_) δ 166.8, 139.7, 137.8, 135.6, 132.9, 129.6, 129.4, 127.5, 127.4, 126.3, 125.3, 95.9, 51.9. HRMS (TOF): m/z calcd for C_34_H_26_N_2_O_4_: 527.19653 found: 527.19705 (M + H)^+^.

Dimethyl 4,4’-(1,4-bis(4-fluorophenyl)-1,4-dihydropyrrolo[3,2-b]pyrrole-2,5-diyl)dibenzoate (4b): Pale yellow solid, 72%, mp.: 302–303 ^o^C. IR spectrum, ν, cm^–1^: 3141, 2943, 1718, 1602. ^1^H NMR (500 MHz, CDCl_3_) δ 7.88 (s, 4 H), 7.39–7.28 (s, 14 H), 6.51 (s, 2 H), 3.89 (s, 6 H). ^13^C NMR (75 MHz, DMSO) δ 166.1, 161.8, 158.5, 147.5, 136.7, 135.1, 134.9, 132.1, 128.9, 126.8, 126.3, 126.1, 115.8, 115.5, 94.9, 51.4. HRMS (TOF): m/z calcd for C_34_H_24_N_2_F_2_O_4_: 563.17769 found: 563. 17,532 (M + H)^+^.

Dimethyl 4,4’-(1,4-bis(4-acetylphenyl)-1,4-dihydropyrrolo[3,2-b]pyrrole-2,5-diyl)dibenzoate (4c): Yellow solid, 68%, mp.:156–157 ^o^C. IR spectrum, ν, cm^–1^: 3106, 1713, 1677, 1598. ^1^H NMR (500 MHz, CDCl_3_) δ 7.99 (d, *J* = 8.0 Hz, 4 H), 7.93 (d, *J* = 7.8 Hz, 4 H), 7.34 (d, *J* = 7.9 Hz, 4 H), 7.28 (d, *J* = 8.3 Hz, 4 H), 6.57 (s, 2 H), 3.91 (s, 6 H), 2.62 (s, 6 H), ^13^C NMR (126 MHz, CDCl_3_) δ 197.0, 166.8, 143.4, 137.3, 135.7, 134.5, 132.5, 129.8, 129.8, 128.1, 127.7, 124.7, 97.5, 52.2, 26.4. HRMS (TOF): m/z calcd for C_38_H_30_N_2_O_6_: 611.21766 found: 611.21516 (M + H)^+^.

### General Synthesis of A-D-A System (5a-b)

Among the synthesized TAPP structures, the formylation of 3,6 positions of compound **4a-c** was carried out by classical Vilsmeier-Haack reaction. 7.5 mL DMF was taken into a two-necked reaction flask and filled with inert gas. Then, this mixture was cooled to 0 °C in an ice bath, 6 mL POCl_3_ compound was slowly added to the cooled reaction flask and stirred for 2 h. Then, compound **4a-c(**0.5 mmol**)** dissolved in 10 mL DMF was added dropwise to this mixture and the mixture was stirred at 80 °C for 8 h. At the end of 8 h, the mixture was cooled, poured into ice water, and neutralized with sodium bicarbonate. The solution mixture was extracted with DCM, and the organic phase was dried, and the solvent was evaporated. The resulting mixture was crystallized in MeOH [27] (Scheme 1).

Dimethyl 4,4’-(3,6-diformyl-1,4-diphenyl-1,4-dihydropyrrolo[3,2-b]pyrrole-2,5-diyl)dibenzoate (5a): Yellow-off solid, 54%, mp.:302–304 ^o^C. IR spectrum, ν, cm^–1^: 2961, 2843, 2789, 1713, 1682, 1607. ^1^H NMR (400 MHz, CDCl_3_) δ 9.40 (s, 2 H), 7.87 (d, *J* = 8.5 Hz, 4 H), 7.33–7.27 (m, 6 H), 7.25 (d, *J* = 8.5 Hz, 4 H), 7.18 (dd, *J* = 7.4, 2.1 Hz, 4 H), 3.83 (s, 6 H). ^13^C NMR (126 MHz, CDCl_3_) δ 184.1, 166.4, 145.7, 138.0, 134.0, 131.6, 130.3, 129.2, 128.7, 128.5, 128.4, 128.3, 110.9, 52.2.

Dimethyl 4,4’-(1,4-bis(4-fluorophenyl)-3,6-diformyl-1,4-dihydropyrrolo[3,2-b]pyrrole-2,5-diyl)dibenzoate (5b): White-off solid, 89%, mp.: 341–342 ^o^C. IR spectrum, ν, cm^–1^: 2960, 2821, 2751, 1717, 1665. ^1^H NMR (400 MHz, CDCl_3_) δ 9.39 (s, 2 H), 7.90 (d, *J* = 8.2 Hz, 4 H), 7.24 (d, *J* = 8.2 Hz, 4 H), 7.17–7.11 (m, 4 H), 6.96 (t, *J* = 7.5 Hz, 4 H), 3.84 (s, 6 H). ^13^C NMR (126 MHz, CDCl_3_) δ 183.2, 165.2, 161.3 (d, *J* = 249.2 Hz), 145.2, 132.9, 132.9, 132.6, 130.5, 129.5, 129.1 (d, *J* = 9.0 Hz), 128.3, 127.2, 114.5 (d, *J* = 23.0 Hz), 51.4. HRMS (TOF): m/z calcd for C_36_H_24_N_2_F_2_O_6_: 619.16752 found: 619.16463 (M + H)^+^.

## Results and Discussion

### Chemistry and Characterization

**Fig. 1:**
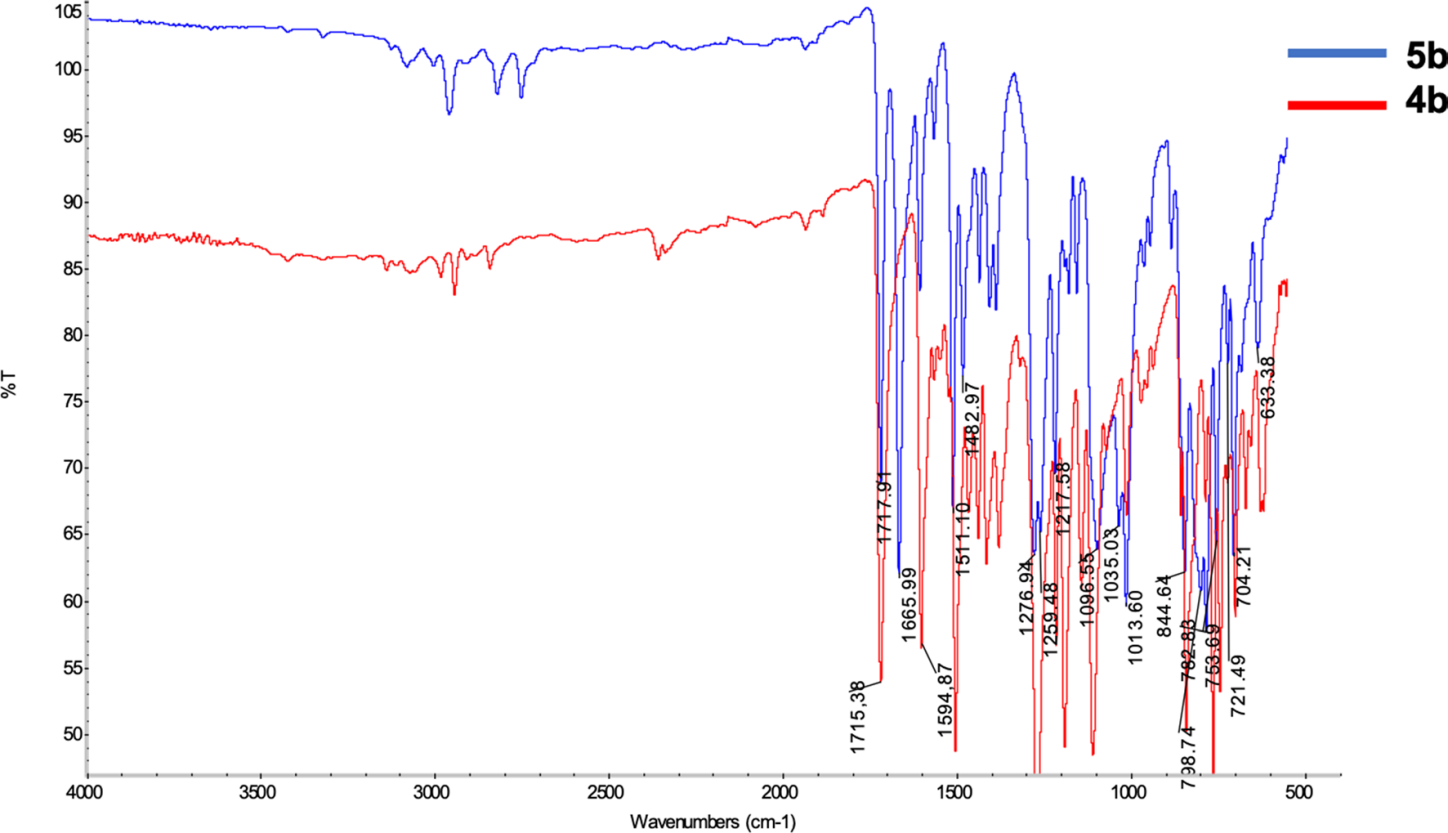
FTIR spetrum of **4b** and **5b**

**Fig. 2:**
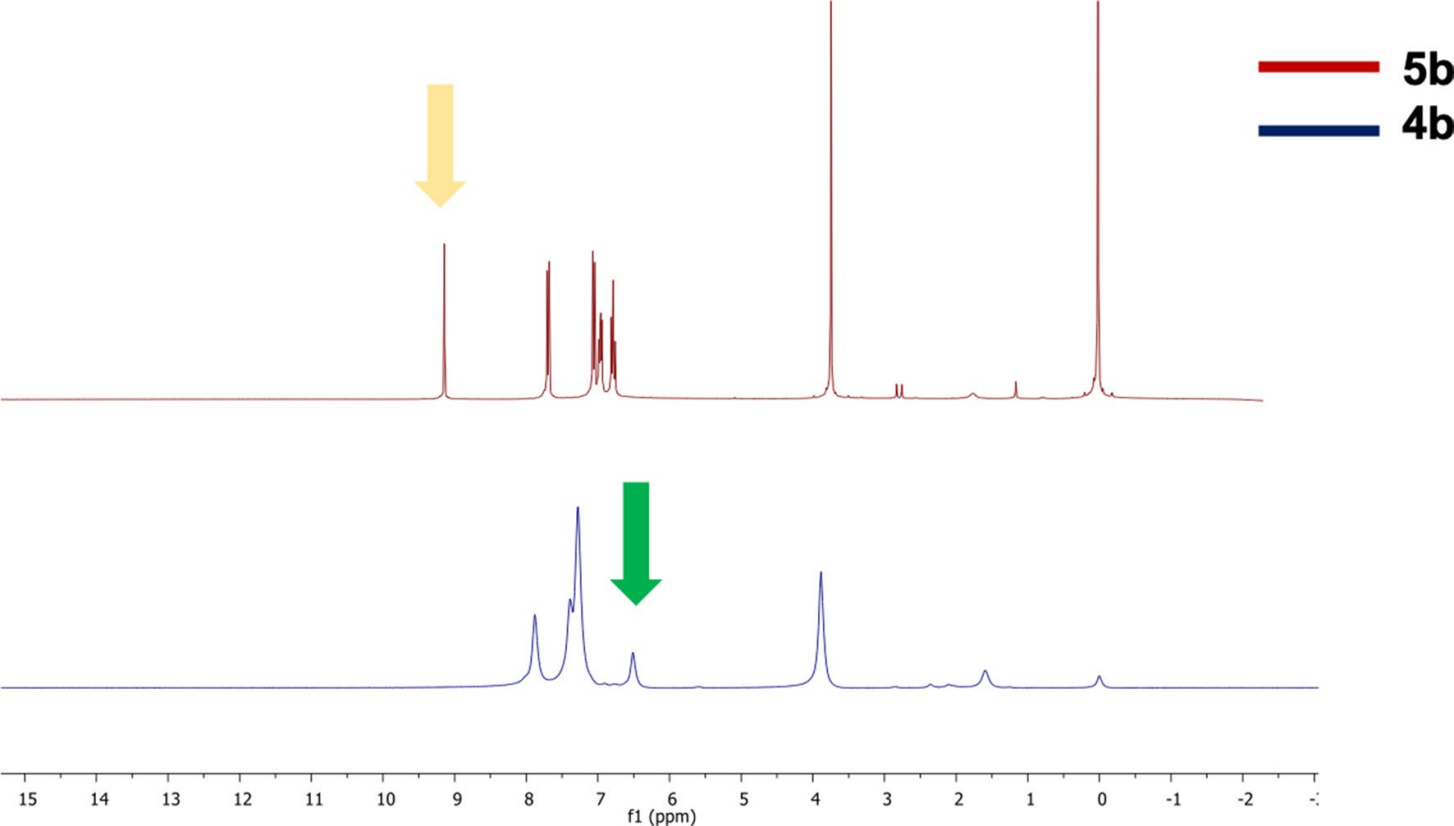
^1^H NMR spectrum of **4b** and **5b**

The aim of this study encompasses the synthesis, characterization, and determination of the photophysical properties of different substituents, especially in *N*-phenyl rings of a series of new TAPP structures that have not been synthesized before. For this purpose, the synthesis of TAPP structures was achieved in a single step with the previously known synthesis method [9]. All obtained TAPPs molecules were subjected to Vilsmeier Haack reaction to attempt to form the targeted A-D-A systems. Among these TAPPs, **4a** and **4b** derivatives, **5a** and **5b** derivatives were obtained with 54% and 89% yield, respectively. For the formylation of compound **5c**, labeled as a derivative containing acetyl group at *N*-phenyl ring, studies with the same conditions or different approaches were attempted but this reaciton was failed given that carbonyl compounds lead to multiple iminoalkylations in Vilsmeier-Haack reaction and the resulting intermediates undergo cyclization to afford aromatic or heterocyclic compounds in the literature [28–29]. Compound **4a-c**,** 5a-b** structures were elucidated using ^1^H NMR, Mass, and ^13^C NMR techniques. Both ^1^H NMR and ^13^C NMR spectroscopy were used to characterize TAPP structures. The H and C peaks at position 3,6, which are specific peaks for TAPPs, appeared around 6.5 and 100 ppm in ^1^H NMR and ^13^C NMR, respectively. During the conversion of compound **4b** to compound **5b**, these specific H peaks disappeared around 6.5 ppm and formyl peaks appeared around 10.5 ppm in ^1^H NMR. Furthermore, the C = O stretching band belonging to the formyl groups in TAPP, **5b**, was observed at 1665 cm^− 1^ in the FTIR spectrum. Finally, the structure of compound number **5b** was completely confirmed with the disappearance of the C peak of the C = O group belonging to aldehydes around 190 ppm in ^13^C NMR spectrum. HRMS analysis results of all synthesized compounds agreed with other techniques and were detected with deviations around +/- 5 ppm. (see Figs. 1 & 2).

### Analysis of Optoelectronic Properties

**Table 1:** Photophysical properties of derivatives **4c** in toluene; **4a-b** obtained in toluene, THF and **5a-b** in obtained THF, DCM

Comp.	Solvent	λ_max (Ab)_ (nm)	ε @ λ_max_ (M^− 1^cm^− 1^)	λ_max (Em)_ (nm)	Stokes shift (cm^− 1^)	Φ_fl._
**4a**	Tol	398	18.716	442	2501	0.24^a^
	THF	397	16.400	441	2513	0.33 ^a^
**4b**	Tol	400	24.636	446	2628	0.41^a^
	THF	397	21.510	455	3210	0.34^a^
**4c**	Tol	396	14.498	441	2576	0.37^a^
**5a**	THF	353	7800	437	5445	0.08^b^
	DCM	352	8100	436	5473	0.11^b^
**5b**	THF	350	8900	439	5792	0.28^b^
	DCM	351	9700	440	5762	0.21^b^

^a^Standard: Coumarin 143 in EtOH (Φfl = 0.54) ^b^Standard: Quinine Sulfate in H_2_SO_4_ (0.5 M Φfl = 0.54)

**Fig. 3:**
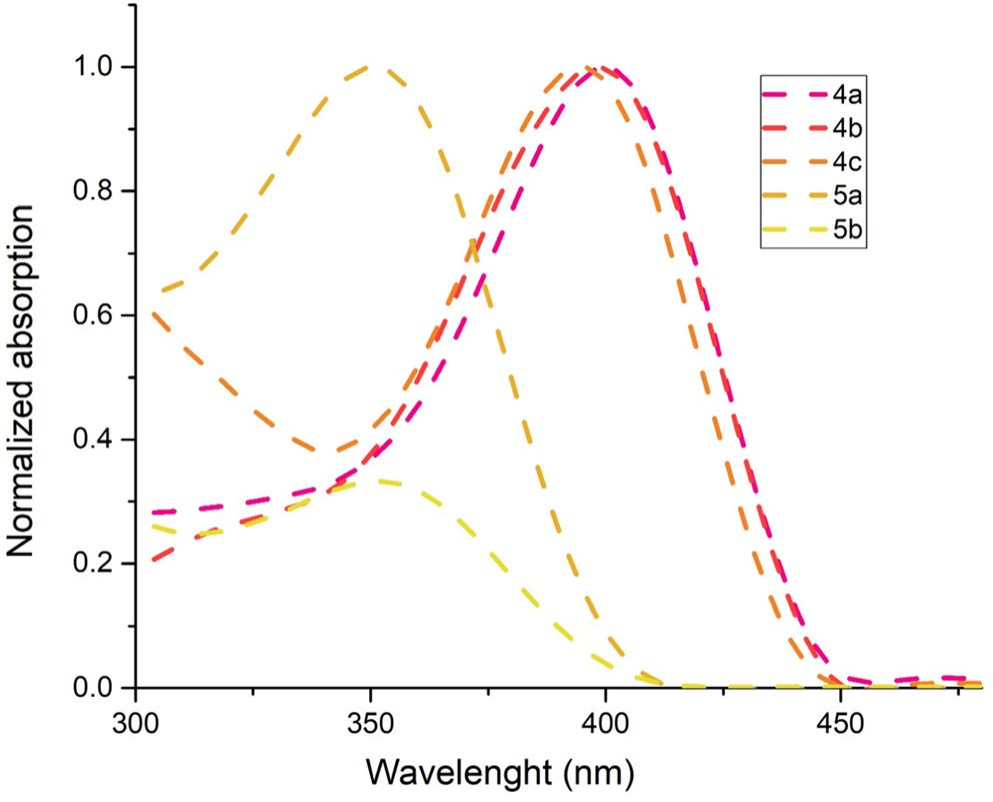
UV-vis spectra of derivatives **4a-c** and **5a-b**

Ultraviolet–visible (UV-vis) studies were conducted in solutions with different dielectric constants (toluene, THF, DCM, MeOH, and DMSO) at a concentration of approximately 10⁻⁶ M. Both TAPP structures and A-D-A systems could not be solubilized in DMSO and MeOH and their photophysical measurements could not be taken. In addition, the solubility of the derivatives in the A-D-A system could not be achieved in toluene. UV-vis and fluorescence spectra for compounds **4a-c** and **5a-b** were examined only in toluene, THF, and DCM. Absorption and emission measurements for compounds **4a-c** were primarily carried out in toluene under 10⁻⁶ M conditions. The molar absorption coefficients of the compounds were determined as follows: 18.716 and 16.400 M⁻¹cm⁻¹ for compound **4a** in toluene and THF, respectively, 24.636 and 21.510 M⁻¹cm⁻¹ for compound **4b** in toluene and THF, respectively, and 14.498 M⁻¹cm⁻¹ for compound **4c**. The absorption maxima corresponding to S₀ → S₁ excitations were observed at 398 nm, 400 nm, and 396 nm for compounds **4a**,** 4b**, and **4c**, respectively. Similarly, emission maxima were recorded at 442 nm, 446 nm, and 441 nm, respectively (Table 1 and SI). With its electron withdrawing property, when there is an acetyl group on the *N*-phenyl ring, it exhibited a 5 nm hypsochromic effect in absorption compared to its unsubstituted derivative on the *N*-phenyl ring (**4a**). F, which is a strong electron withdrawing atom inductively, is also an important substituent providing electron support to the ring according to the Hammett constant. The fact that the F atom in compound 4b produces a bathochromic effect of up to 5 nm suggests that this is only possible if the F atom provides electrons to the ring electronically [30] (Fig. 3 and SI).

**Fig. 4:**
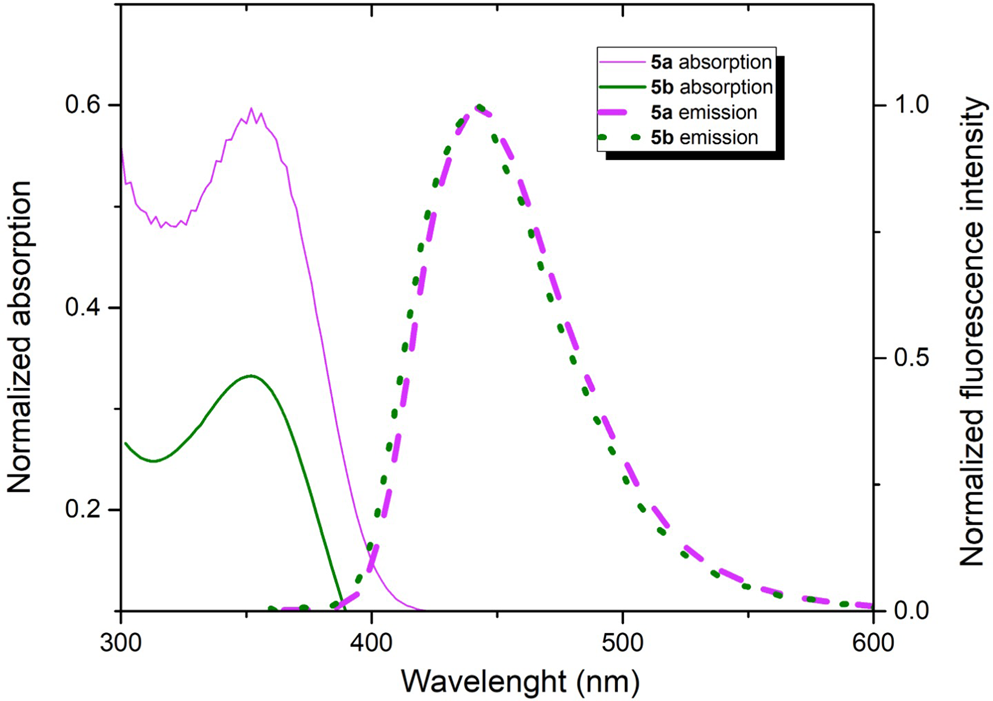
Normalized absorption (solid line) and fluorescence emission spectrum (short-dotted line, excitation at 340 nm of **5a-b**) of compounds **5a** (pink) and **5b** (green) in THF

**Fig. 5:**
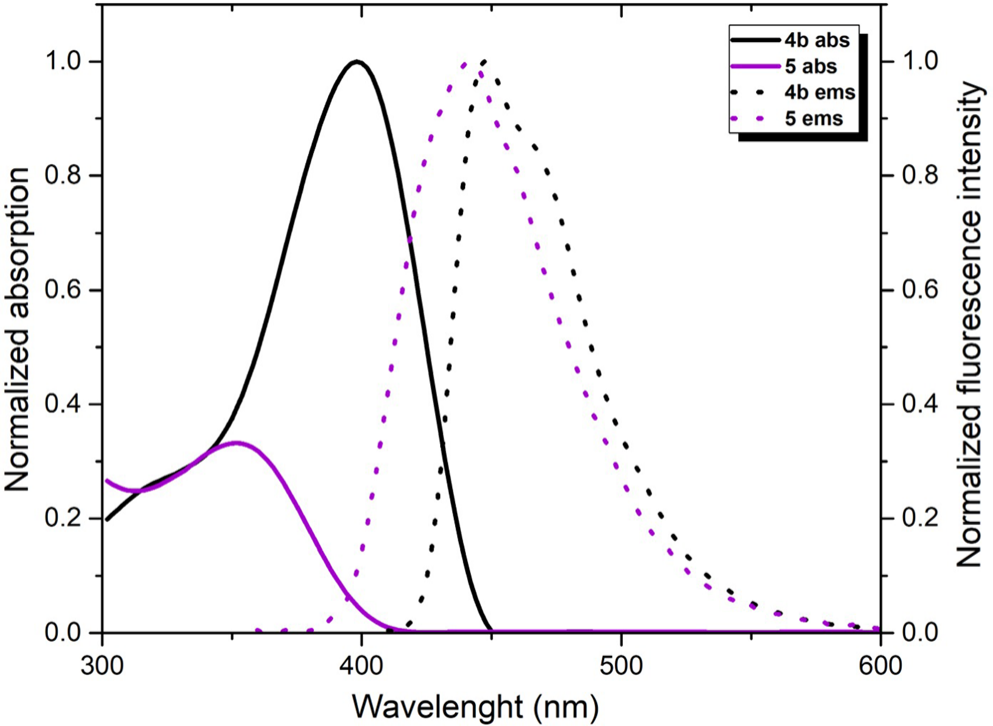
Normalized absorption (solid line) and fluorescence emission spectrum (short-dotted line, excitation at 400 nm of **4b** and excitation at 340 nm of **5b**) of compounds **4b** (black) and **5b** (purple) in THF

As a result of the Vilsmeier-Haack reaction on compound **4a-b**, the photophysical measurements of the resulting compound **5a-b** were conducted in THF and DCM at the same concentration. UV-vis measurements of **5a** and **5b** revealed an absorption maximum around 350 nm with molar absorption coefficients of 7800 and 8900 M⁻¹cm⁻¹ in THF and 8100 and 9700 M⁻¹cm⁻¹ in DCM, respectively, while its emission spectrum showed an emission peak at 440 nm (Table 1 and SI). It was observed that no significant shift occurred in the absorption and emission wavelengths of the formylated products (Table 1; Fig. 4). However, when compared to its precursor compound **4b**, compound **5b** exhibited a significant hypsochromic shift of up to 50 nm in absorption. This shift was attributed to charge transfer caused by the strong electron-withdrawing properties of the formyl groups attached at the 3 and 6 positions. However, it was noted that the emission properties of compound **5b** remained relatively unchanged compared to **4b** (Fig. 5 and SI). **Electron**-withdrawing groups usually pull the HOMO level down, while affecting the LUMO level less or stabilizing it slightly. This is known to lead to an increase in the HOMO-LUMO energy range and a shift to higher energy (shorter wavelength) in the absorption spectrum, i.e. a hypsochromic shift. In the emission process, the molecule undergoes excited-state relaxation. If there is no major rearrangement of the excited state, the emission wavelength may change less compared to absorption. This can cause the emission spectrum to shift less than the absorption spectrum. It is known that fluorophores containing electron-withdrawing and -donating groups undergo intramolecular charge transfer due to excitation by light and consequent changes in the dipole moment leading to Stokes shift. In our study, the electron-withdrawal of formyl structures, which is a mesomerically moderate electron-withdrawing group at the 3,6 positions, is observed with a significant difference in the absorption with the pulling down of the electron at the HOMO levels, while the low molecular rearrangements in the emission are thought to cause the structure with very small emission. Also, the 2,5 positions of the TAPP structures cause the ring to remain in a planarizing form, while the 1,4 positions tend to shift to a twist form with a certain angle to this plane. It is also known from the literature that the binding of different groups or atoms from the 3,6 positions of this ring leads to more pushing and twisting of the structure [31–32]. For these reasons, the bending of the structure with the attachment of formyl groups may have eventually led to a few hypsochromic effect in absorption. The synthesized push-pull (A-D-A) system of compound **5a-b** demonstrated higher quantum efficiency and a more pronounced Stokes shift, consistent with trends observed in similar systems reported in the literature. There are many methods and studies related to TAPP structures in the literature. Most of these studies are provided by modifications on the planar structure from the 2,5-positions but the references to studies with electronic effects at the 1,4-positions are limited. In this study, from this perspective, the aim was to contribute to the literature with different substituents on the *N*-phenyl ring. Furthermore, carbonyl groups are important structures in organic chemistry. Aldehyde, ketone and ester structures, in short, easily modified carbonyl compounds can easily transform into different structures. It is obvious that the synthesized derivatives contain structures belonging to this class, allowing the use of these molecules as precursors in future studies and can be easily derivatized. It is thought that modifications on these carbonyl groups will allow these structures to be adapted to very wide spectrum studies such as OLED, OFET, 2PA, bioimaging [8–13, 21]. It is seen in the literature that A-D-A systems similar to the study we presented have been synthesized. For instance, a study in 2022 highlighted the use of a formylated TAPP structure, similar to compound **5a-b**, as a bioimaging agent, emphasizing the relevance of these structures for molecular imaging [31]. Unlike this study, we believe that the molecules we synthesized are higher than the literature in terms of high efficiency and quantum efficiency and contribute significantly to the synthesis of these molecules. Therefore, it is anticipated that further research on the synthesized molecules will enhance their suitability for advanced optoelectronic and bioimaging applications.

## Conclusion

As a result, 3 new TAPP structures, **4a-c**, and A-D-A type, **5a-b**, structures were introduced to the literature and their structure characterizations were provided. It was observed that there was a difference of about 5 nm in UV-vis/fluorescence measurements according to the electron donating-withdrawing groups at position 4 in structures **4a-c**. When acceptor groups were attached to positions 3,6 of TAPP structure 4b, a hypso shift of about 50 nm was observed in the UV-vis spectrum of the formed **5b** structure, while a 10 nm change was recorded in its fluorometry. We think that the formed pull-push system has a higher Stokes shift compared to compound **4b**, and that **5b** and similar compounds will form the leading structures in 2PA, OLED and many optoelectronic devices. The attachment of aldehyde groups to the 3,6-positions resulted in a pronounced hypsochromic effect in the absorption spectrum of the structure. This effect is due to the electron-withdrawing properties of the aldehyde groups and results in a decrease in the electron density of the molecule, resulting in an increase in the energy of the π-π transitions. The hypsochromic shift generally indicates higher energy light absorption, which can enable the molecule to absorb light effectively at shorter wavelengths, providing more sensitive light sensitivity and selective light absorption properties, especially in optoelectronic and photonic applications. Furthermore, such a modification can further optimize the photophysical properties of the molecule by directing donor-acceptor interactions, allowing for improved performance in processes such as electron transfer.

## Electronic Supplementary Material

Below is the link to the electronic supplementary material.

Supplementary Material 1

## Data Availability

No datasets were generated or analysed during the current study.
