# Supplementary material for: 1,4-Dihydropyrrolo[3,2-b]Pyrroles Containing New A-D-A System: Synthesis and Investigation of Their Photophysical Properties
Source: J Fluoresc. 2025 Mar 21;35(10):9513–20. doi: 10.1007/s10895-025-04240-y (PMC12672777; doi:10.1007/s10895-025-04240-y)
Supplement: Supplementary file 1 — Supplementary Material 1 [file 10895_2025_4240_MOESM1_ESM.docx]

**1,4-Dihydropyrrolo[3,2-b]pyrroles containing new A-D-A system: Synthesis and investigation of their photophysical properties**

Guler YAGIZ ERDEMIR^1^*

^1^Department of Chemistry, Faculty of Science, Gazi University, Ankara, 06560, Turkey

Corresponding Author: Guler YAGIZ ERDEMIR

[*guleryagiz@gazi.edu.tr](mailto:*derya.topkaya@deu.edu.tr)

**TABLE OF CONTENTS**

|  | **Chapter** | **Pages** |
| --- | --- | --- |
|  | **General Experimental Information and Characterization data of products** | **2-3** |
|  | **Copies of FTIR, ^1^H NMR, ^13^C NMR and HRMS spectra of the compounds** | **4-13** |
|  | **Photophysical properties of compounds** | **14-18** |

**General Synthesis of TAPPs (4a-c):**

Aniline (2 mmol), aldehyde (2 mmol), and solvent system (acetic acid/toluene: 2/2 mL) were added to a round-bottomed and wide-mouthed reaction vessel and stirred for 2 hours at 50 ℃. Then, iron (III) perchlorate (catalytic amount) and diacetyl (1 mmol) were added to this mixture and stirred at the same temperature overnight. After this period, 5 mL of ACN was added to the mixture, heated and vibrated, and hot filtration was performed. The solids obtained were washed twice with ACN, and pure TAPP structures were obtained [1, 2].

**Dimethyl 4,4'-(1,4-diphenyl-1,4-dihydropyrrolo[3,2-b]pyrrole-2,5-diyl)dibenzoate (4a):** Yellow solid, 78%, **mp.:** 276 ^o^C**.** **IR spectrum, ν, cm^–1^:** 3105, 2949, 1712, 1598. **^1^H NMR (500 MHz, CDCl_3_) δ** 7.89 (d, *J =* 8.2 Hz, 4H), 7.88 7.39 (t, *J* = 7.6 Hz, 4H), 7.87-7.26 (m, 10H), 6.51 (s, 2H), 3.88 (s, 6H). **^13^C NMR (126 MHz, CDCl_3_) δ** 166.8, 139.7, 137.8, 135.6, 132.9, 129.6, 129.4, 127.5, 127.4, 126.3, 125.3, 95.9, 51.9. **HRMS (TOF): m/z** calcd for C_34_H_26_N_2_O_4_: 527.19653 found: 527.19705 (M+H)^+^.

**Dimethyl 4,4'-(1,4-bis(4-fluorophenyl)-1,4-dihydropyrrolo[3,2-b]pyrrole-2,5-diyl)dibenzoate (4b):** Pale yellow solid, 72%, **mp.:** 302-303 ^o^C. **IR spectrum, ν, cm^–1^:** 3141, 2943, 1718, 1602. **^1^H NMR (500 MHz, CDCl_3_) δ** 7.88 (s, 4H), 7.39-7.28 (s, 14H), 6.51 (s, 2H), 3.89 (s, 3H). **^13^C NMR (75 MHz, DMSO) δ** 166.1, 161.8, 158.5, 147.5, 136.7, 135.1, 134.9, 132.1, 128.9, 126.8, 126.3, 126.1, 115.8, 115.5, 94.9, 51.4. **HRMS (TOF): m/z** calcd for C_34_H_24_N_2_F_2_O_4_: 563.17769 found: 563. 17532 (M+H)^+^.

**Dimethyl 4,4'-(1,4-bis(4-acetylphenyl)-1,4-dihydropyrrolo[3,2-b]pyrrole-2,5-diyl)dibenzoate (4c):** Yellow solid, 68%, **mp.:**156-157 ^o^C. **IR spectrum, ν, cm^–1^:** 3106, 1713, 1677, 1598. **^1^H NMR (500 MHz, CDCl_3_) δ** 7.99 (d, *J* = 8.0 Hz, 4H), 7.93 (d, *J* = 7.8 Hz, 4H), 7.34 (d, *J* = 7.9 Hz, 4H), 7.28 (d, *J* = 8.3 Hz, 4H), 6.57 (s, 2H), 3.91 (s, 6H), 2.62 (s, 6H), **^13^C NMR (126 MHz, CDCl_3_) δ** 197.0, 166.8, 143.4, 137.3, 135.7, 134.5, 132.5, 129.8, 129.8, 128.1, 127.7, 124.7, 97.5, 52.2, 26.4. **HRMS (TOF): m/z** calcd for C_38_H_30_N_2_O_6_: 611.21766 found: 611.21516 (M+H)^+^

**Synthesis of dimethyl 4,4'-(1,4-bis(4-fluorophenyl)-3,6-diformyl-1,4-dihydropyrrolo[3,2-b]pyrrole-2,5-diyl)dibenzoate (5)**

Among the synthesized TAPP structures, the formylation of 3,6 positions of compound **4b** was carried out by classical Vilsmeier-Haack reaction. 7.5 mL DMF was taken into a two-necked reaction flask and filled with inert gas. Then, this mixture was cooled to 0 °C in an ice bath, 6 mL POCl_3_ compound was slowly added to the cooled reaction flask and stirred for 2 h. Then, compound **4b** dissolved in 10 mL DMF was added dropwise to this mixture and the mixture was stirred at 80 °C for 8 h. At the end of 8 h, the mixture was cooled, poured into ice water, and neutralized with sodium bicarbonate. The solution mixture was extracted with DCM, and the organic phase was dried, and the solvent was evaporated. The resulting mixture was crystallized in MeOH [3].

**Dimethyl 4,4'-(3,6-diformyl-1,4-diphenyl-1,4-dihydropyrrolo[3,2-b]pyrrole-2,5-diyl)dibenzoate (5a)**: Yellow-off solid, 54%, **mp.:**302-304 ^o^C. **IR spectrum, ν, cm^–1^:** 2961, 2843, 2789, 1713, 1682, 1607. **^1^H NMR (400 MHz, CDCl_3_) δ** 9.40 (s, 2H), 7.87 (d, *J* = 8.5 Hz, 4H), 7.33-7.27 (m, 6H), 7.25 (d, *J* = 8.5 Hz, 4H), 7.18 (dd, *J* = 7.4, 2.1 Hz, 4H), 3.83 (s, 6H). **^13^C NMR (126 MHz, CDCl_3_) δ** 184.1, 166.4, 145.7, 138.0, 134.0, 131.6, 130.3, 129.2, 128.7, 128.5, 128.4, 128.3, 110.9, 52.2.

**Dimethyl 4,4'-(1,4-bis(4-fluorophenyl)-3,6-diformyl-1,4-dihydropyrrolo[3,2-b]pyrrole-2,5-diyl)dibenzoate (5b):** White-off solid, 89%, **mp.:** 341-342 ^o^C. **IR spectrum, ν, cm^–1^:** 2960, 2821, 2751, 1717, 1665. **^1^H NMR (400 MHz, CDCl_3_) δ** 9.39 (s, 2H), 7.90 (d, *J* = 8.2 Hz, 4H), 7.24 (d, *J* = 8.2 Hz, 4H), 7.17-7.11 (m, 4H), 6.96 (t, *J* = 7.5 Hz, 4H), 3.84 (s, 6H). **^13^C NMR (126 MHz, CDCl_3_) δ** 183.2, 165.2, 161.3 (d, *J* = 249.2 Hz), 145.2, 132.9, 132.9, 132.6, 130.5, 129.5, 129.1 (d, *J* = 9.0 Hz), 128.3, 127.2, 114.5 (d, *J* = 23.0 Hz), 51.4. **HRMS (TOF): m/z** calcd for C_36_H_24_N_2_F_2_O_6_: 619.16752 found: 619.16463 (M+H)^+^

**Scheme 1**. Synthesis of the target molecules

**Copies of FTIR, ^1^H NMR, ^13^C NMR and HRMS spectra of the compounds**


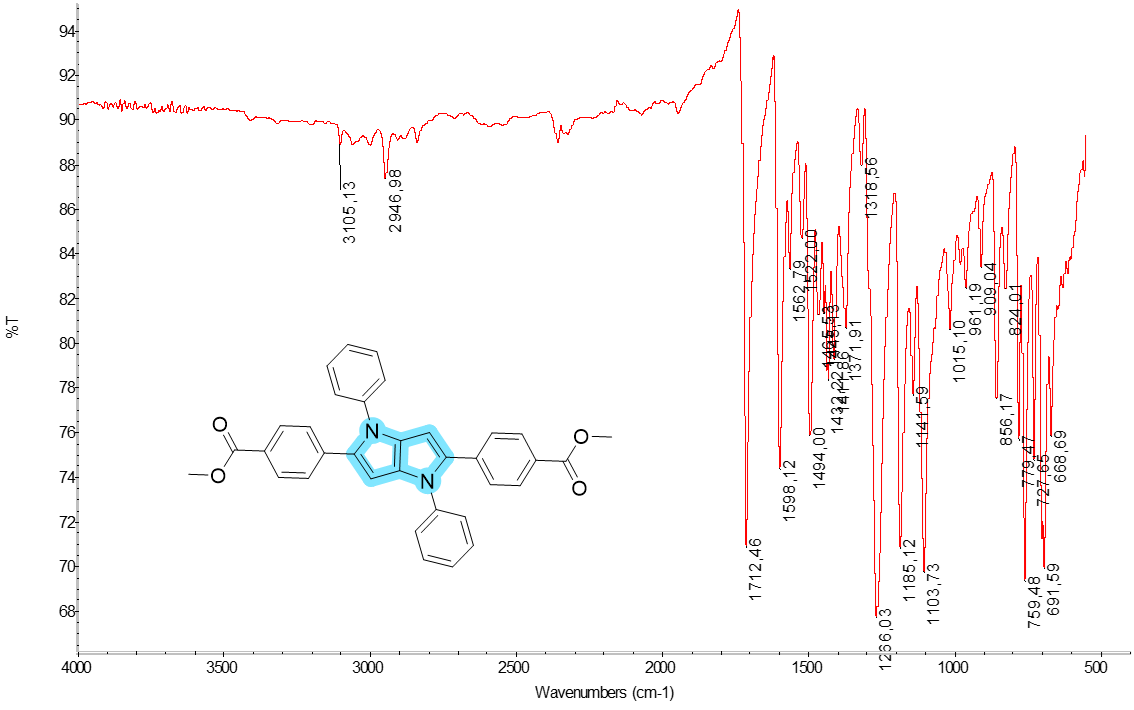


**Figure S1**. FTIR spectra of **4a**


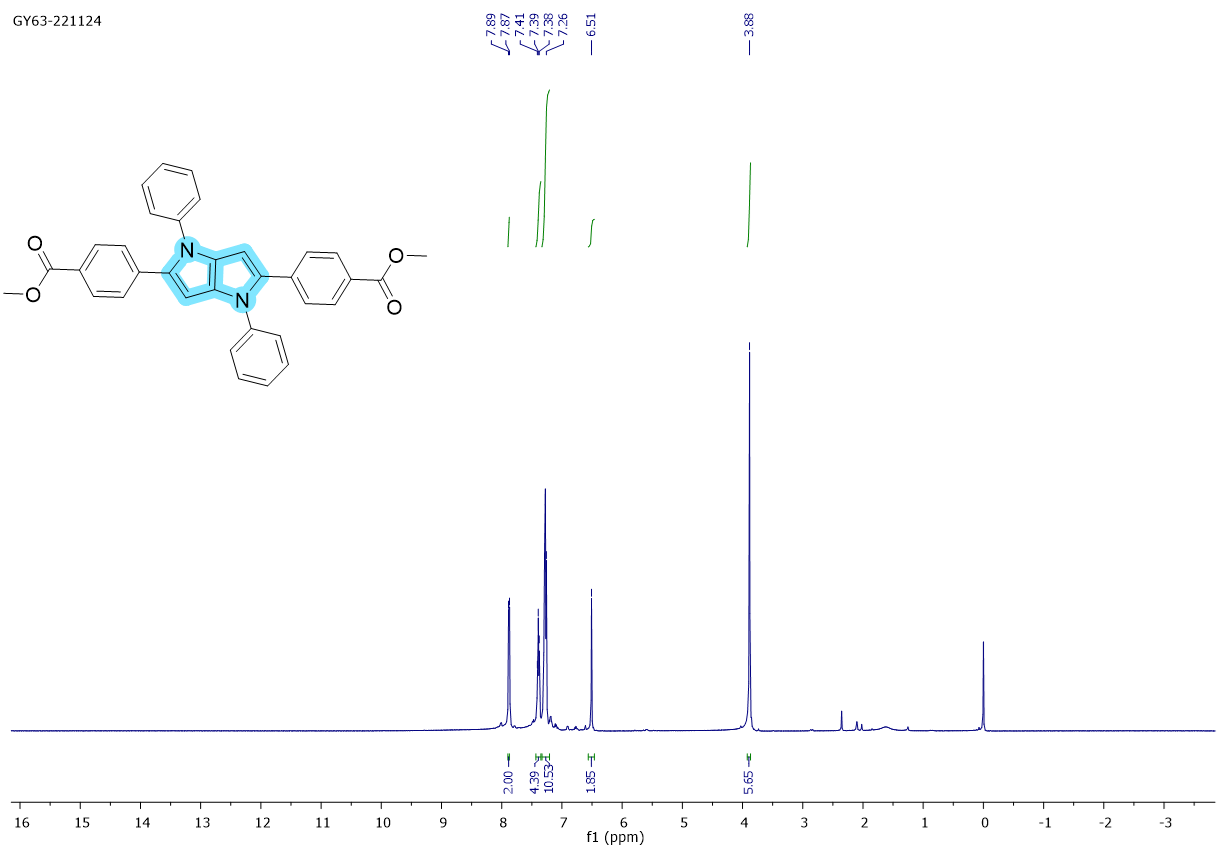


**Figure S2**. ^1^H NMR spectra of **4a**


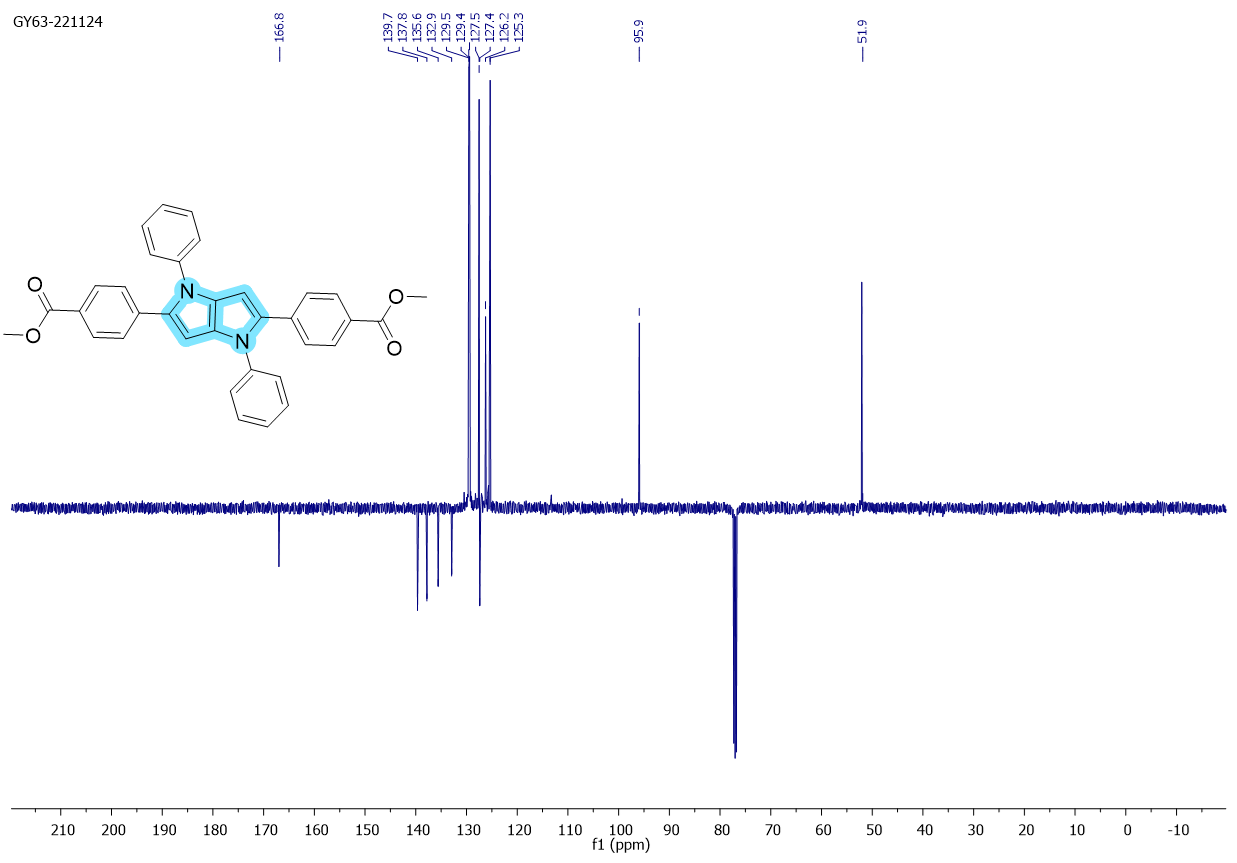


**Figure S3**. ^13^C NMR spectra of **4a**


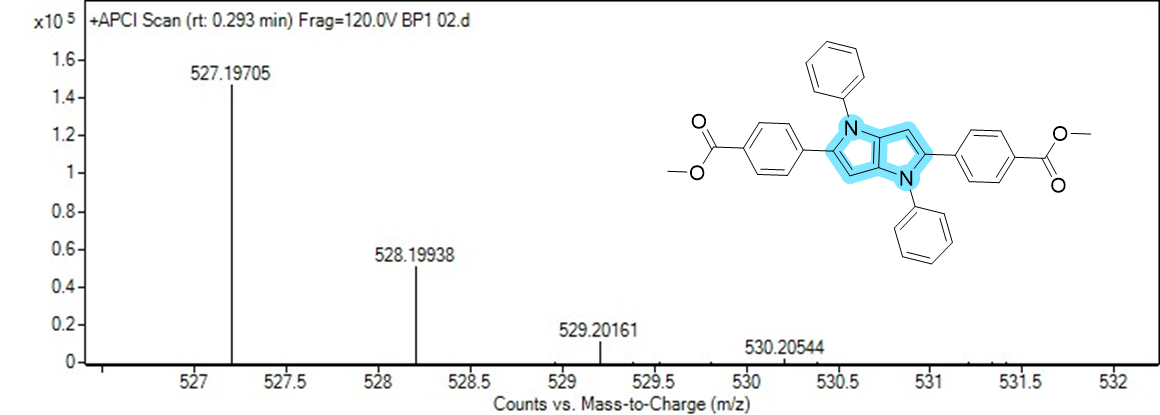


**Figure S4**. HRMS spectra of **4a**


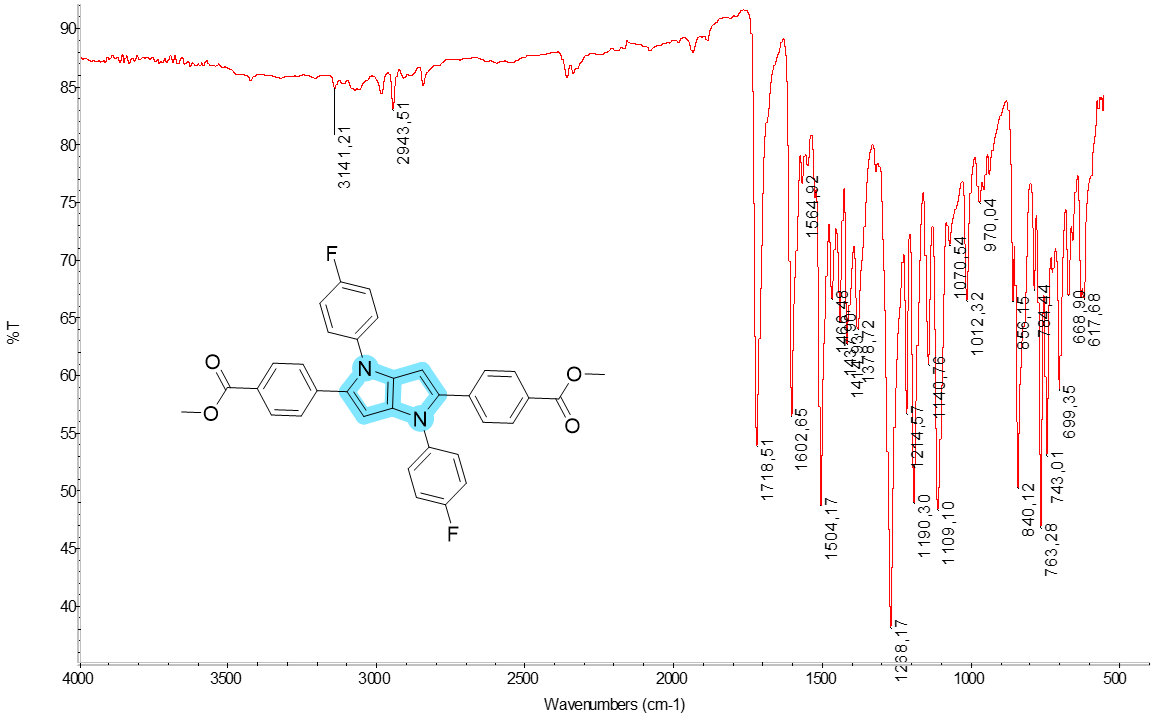


**Figure S5**. FTIR spectra of **4b**


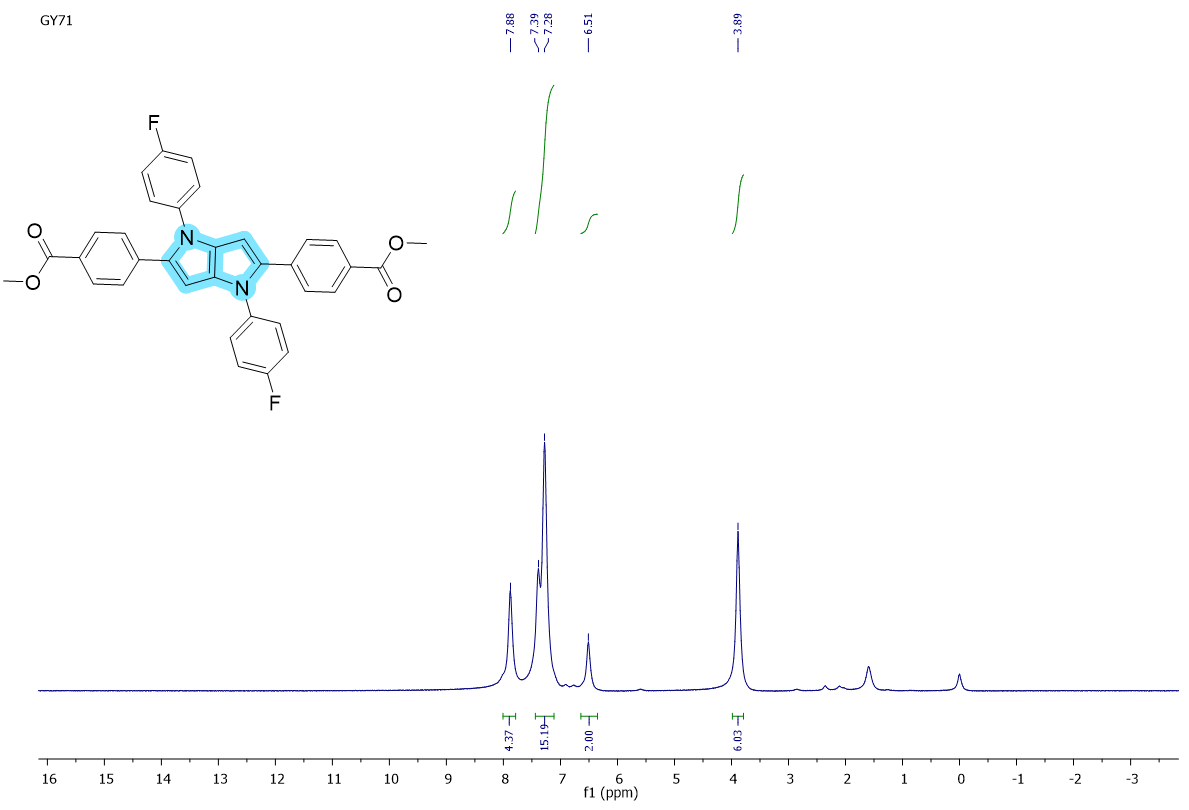


**Figure S6**. ^1^H NMR spectra of **4b**


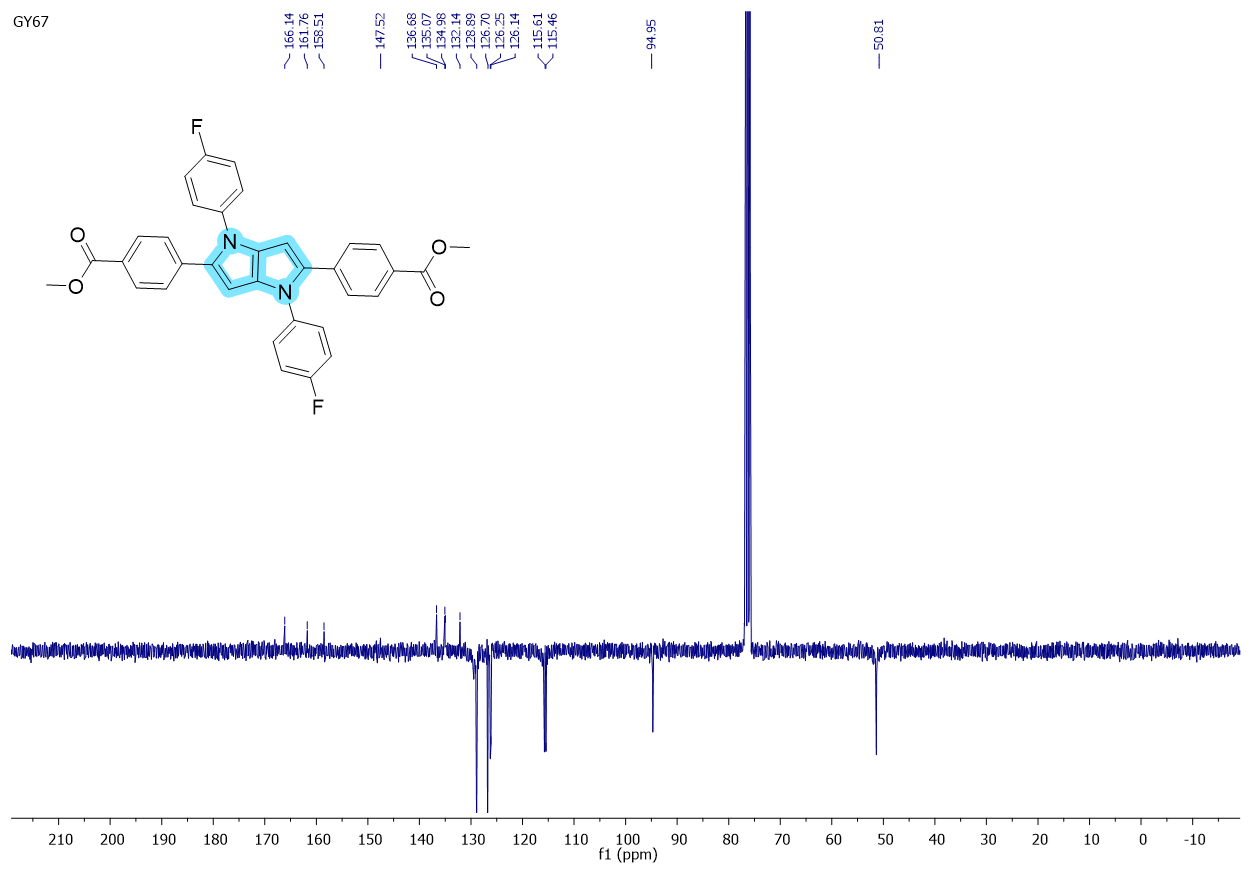


**Figure S7**. ^13^C NMR spectra of **4b**


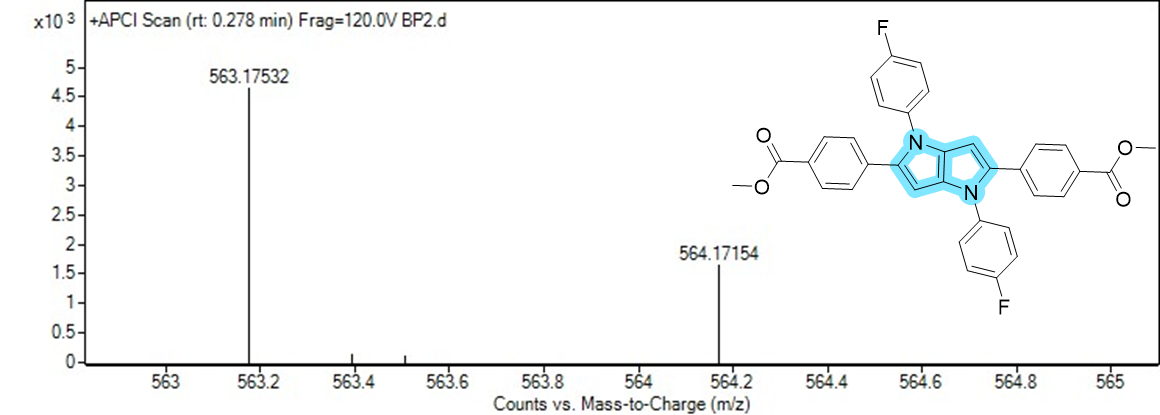


**Figure S8**. HRMS spectra of **4b**


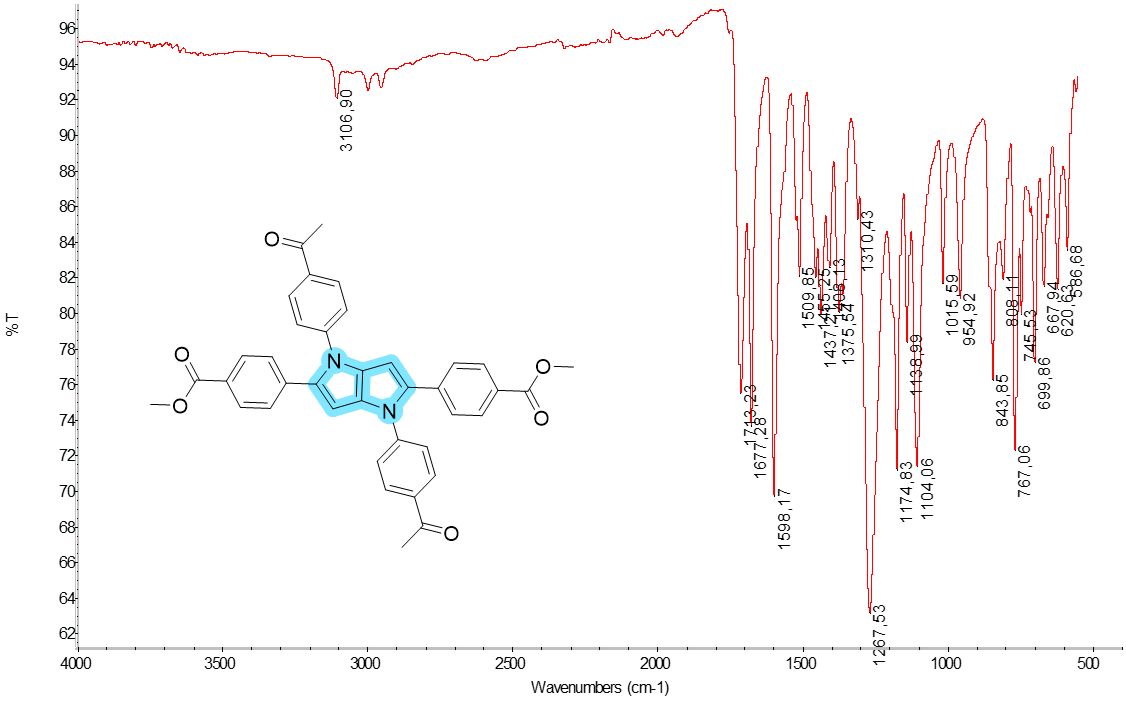


**Figure S9**. FTIR spectra of **4c**


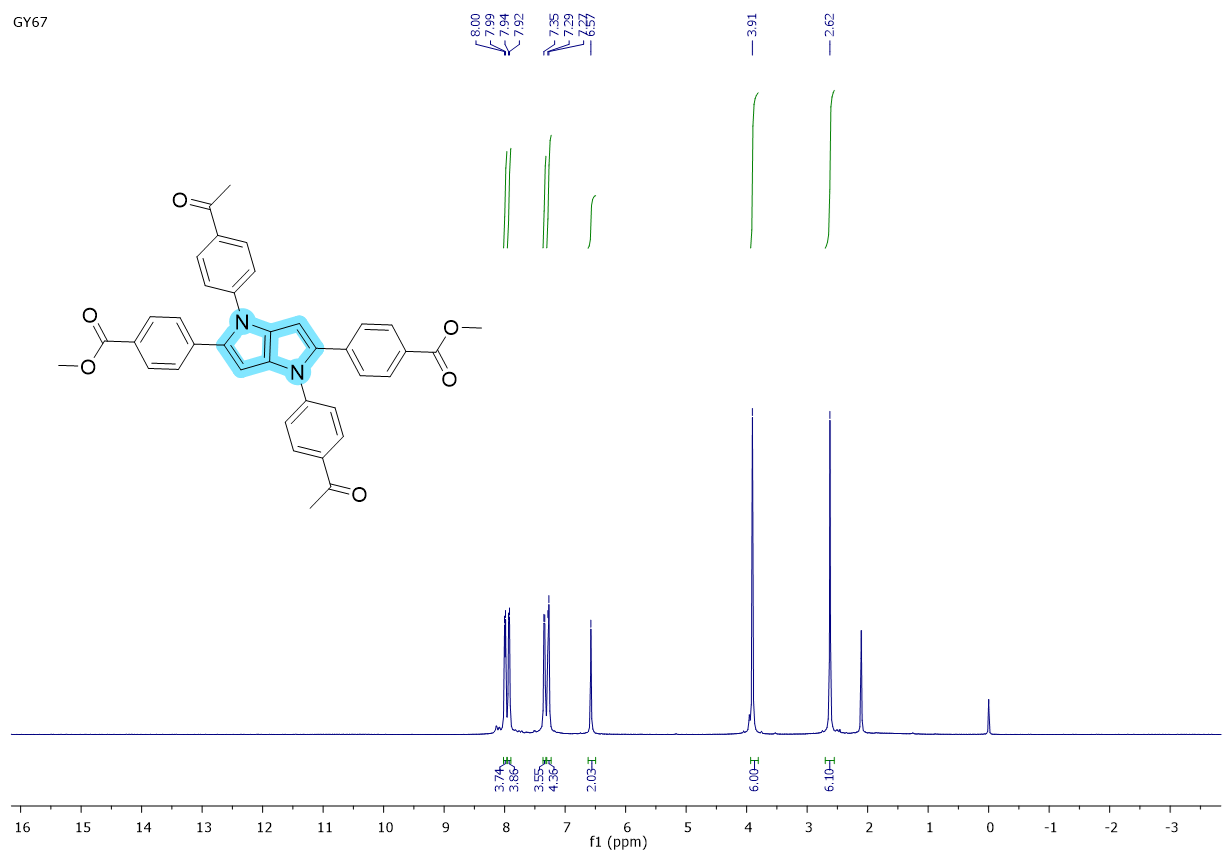


**Figure S10**. ^1^H NMR spectra of **4c**


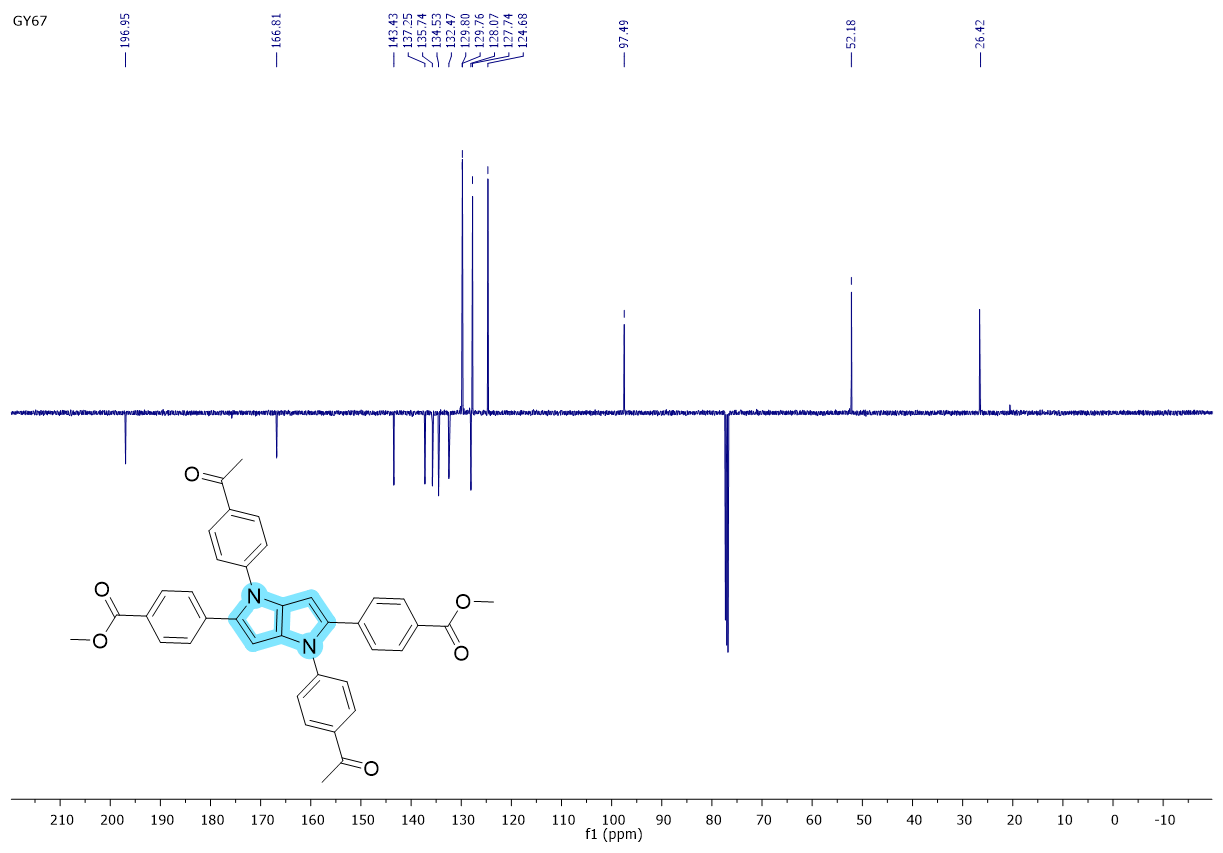


**Figure S11**. ^13^C NMR spectra of **4c**


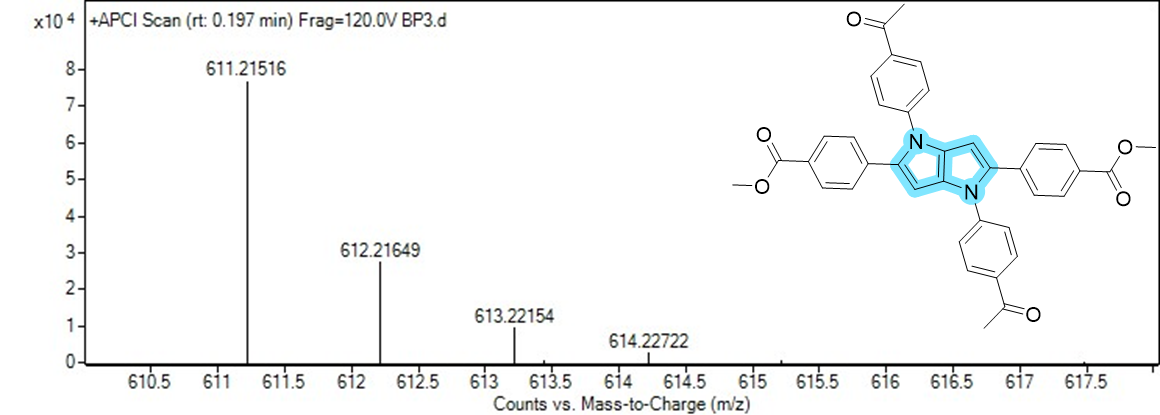


**Figure S12**. HRMS spectra of **4c**

**Figure S13**. FTIR spectra of **5a**

**Figure S14**. ^1^H NMR spectra of **5a**

**Figure S15**. ^13^C NMR spectra of **5a**


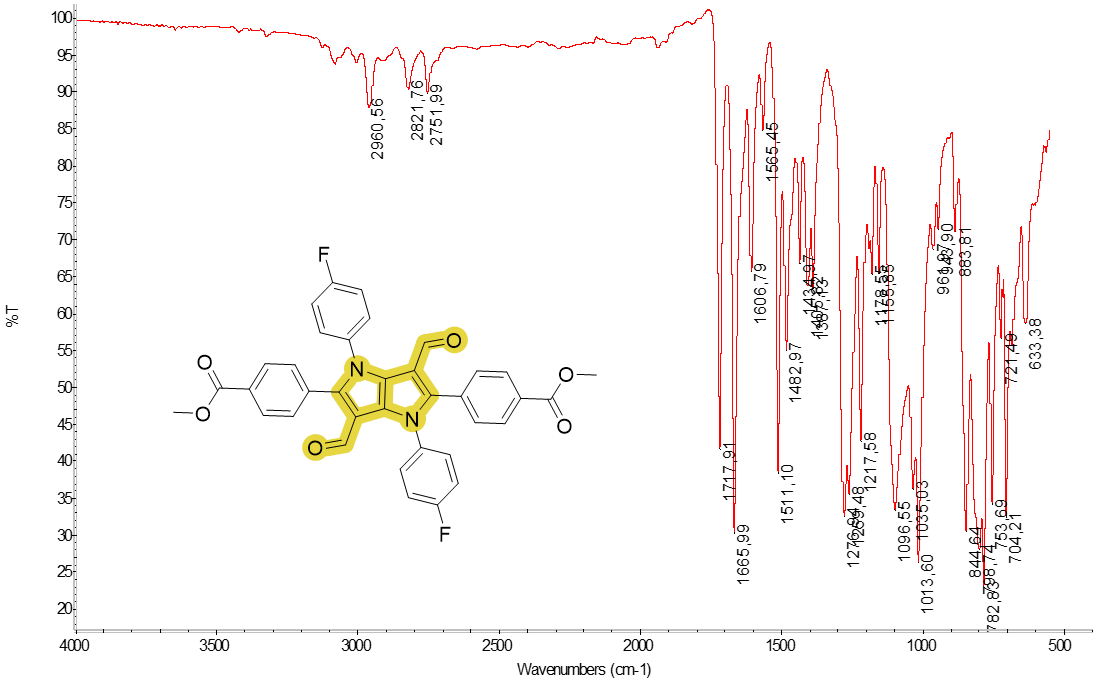


**Figure S16**. FTIR spectra of **5b**


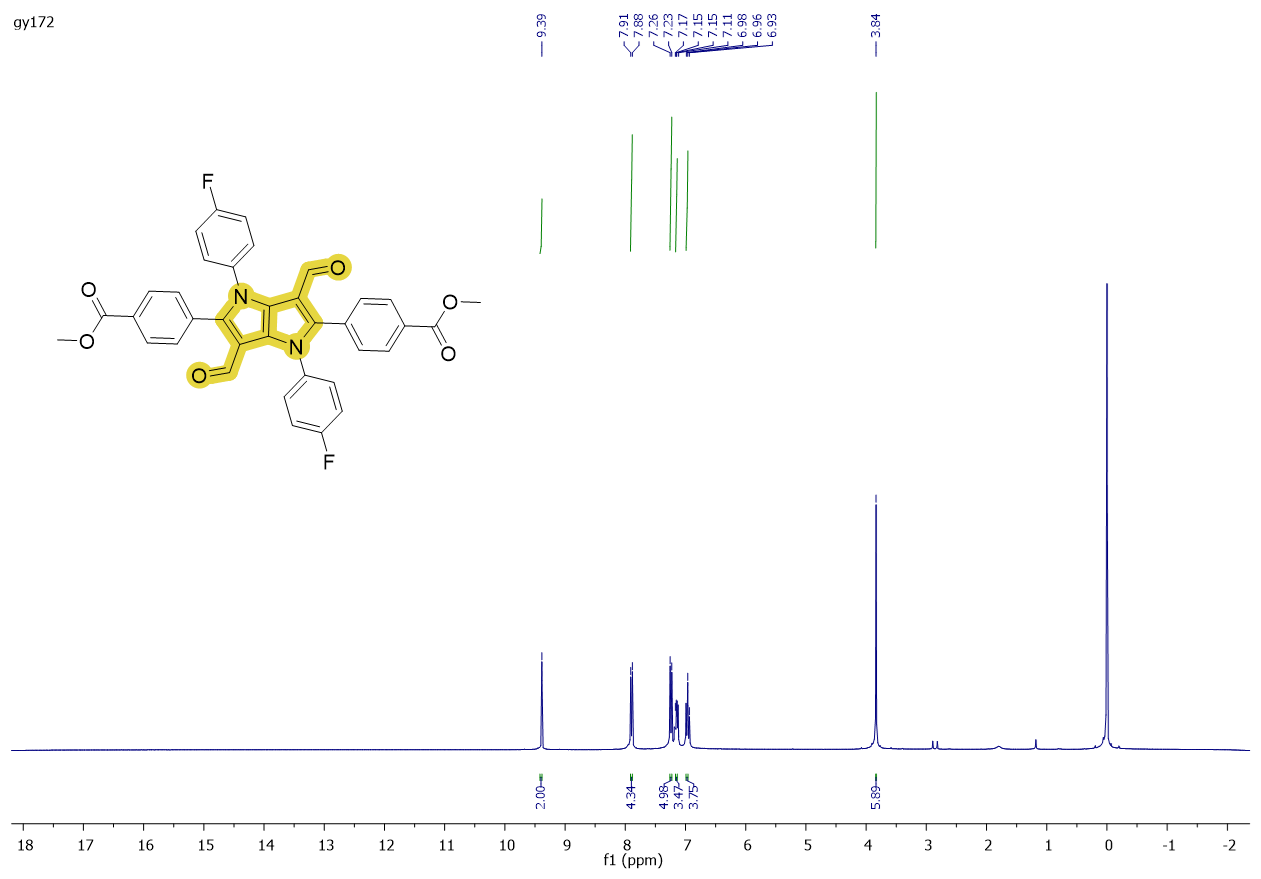


**Figure S17**. ^1^H NMR spectra of **5b**


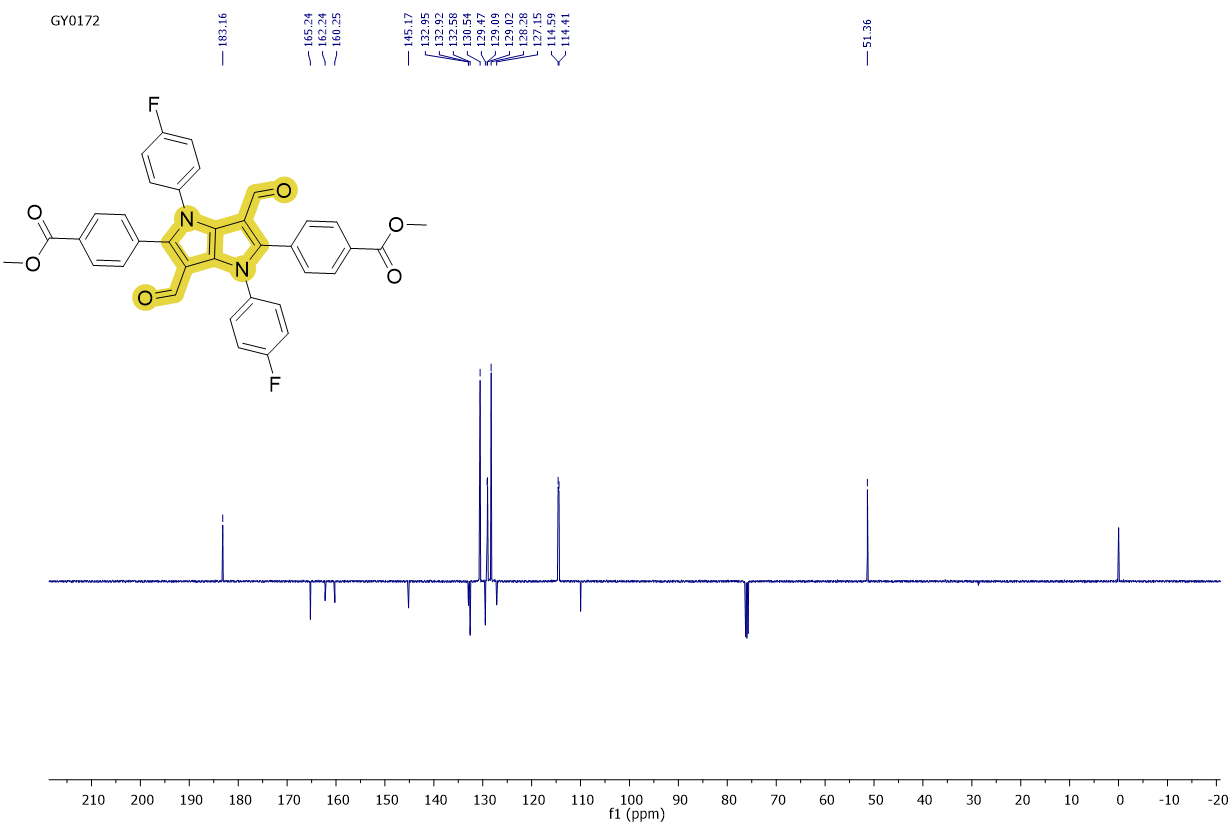


**Figure S18**. ^13^C NMR spectra of **5b**


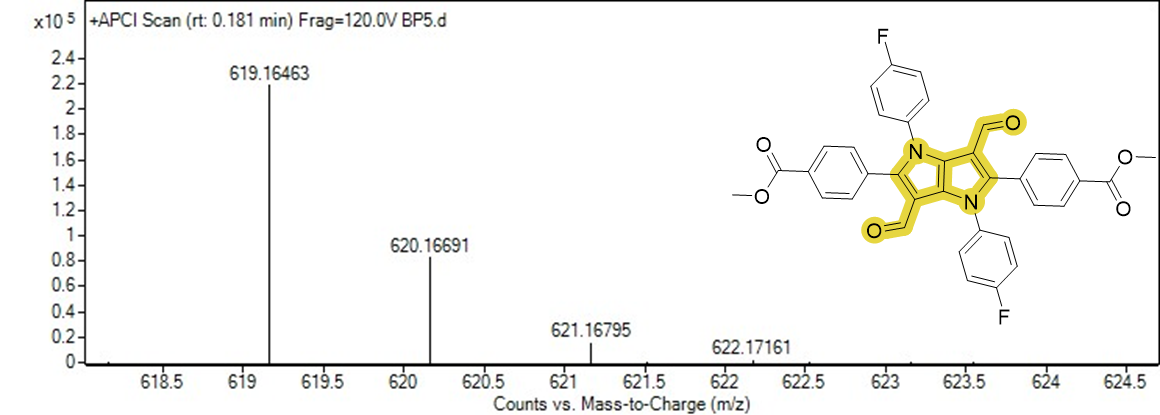


**Figure S19**. HRMS spectra of **5b**

**Photophysical properties of compounds**

**Table 1.** Photophysical Properties of Derivatives **4c** in Toluene; **4a-b** Obtained in Toluene, THF and **5a-b** in Obtained THF, DCM

| **Comp.** | **Solvent** | **λ_max (Ab)_**  **(nm)** | ***ε* @ λ_max_**  **(M^-1^cm^-1^)** | **λ_max (Em)_**  **(nm)** | **Stokes Shift**  **(cm^-1^)** | ***Φ*_fl_** |
| --- | --- | --- | --- | --- | --- | --- |
| **4a** | Tol | 398 | 18716 | 442 | 2501 | 0.24^a^ |
|  | THF | 397 | 16400 | 441 | 2513 | 0.33 ^a^ |
| **4b** | Tol | 400 | 24636 | 446 | 2628 | 0.41^a^ |
|  | THF | 397 | 21510 | 455 | 3210 | 0.34^a^ |
| **4c** | Tol | 396 | 14498 | 441 | 2576 | 0.37^a^ |
| **5a** | THF | 353 | 7800 | 437 | 5445 | 0.08^b^ |
|  | DCM | 352 | 8100 | 436 | 5473 | 0.11^b^ |
| **5b** | THF | 350 | 7800 | 439 | 5792 | 0.28^b^ |
|  | DCM | 351 | 8100 | 440 | 5762 | 0.21^b^ |

^a^Standard: Coumarin 143 in EtOH (Φfl = 0.54) ^b^Standard: Quinine Sulfate in H_2_SO_4_ (0.5 M Φfl = 0.54).


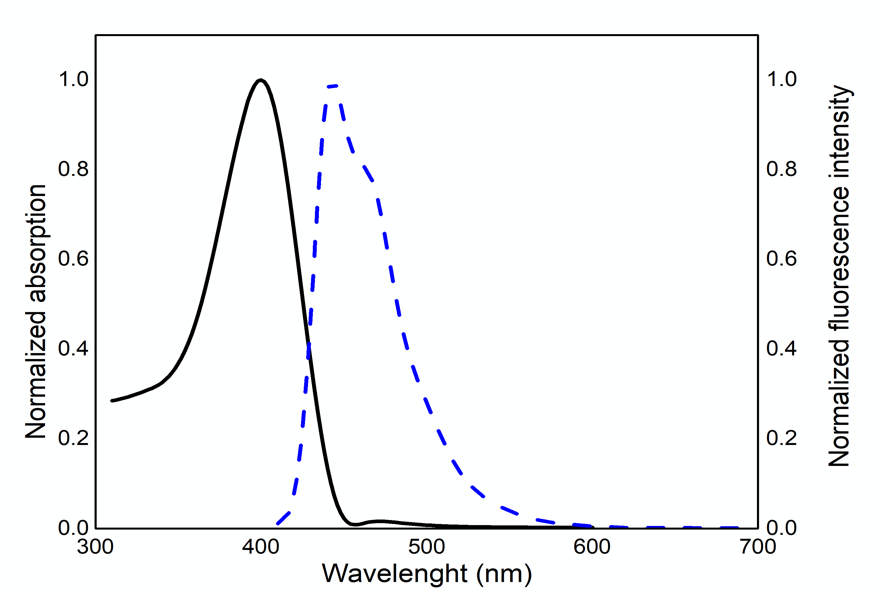


**Figure S20.** UV-Vis and Fluorescence Spectra of Compound **4a** in Toluene


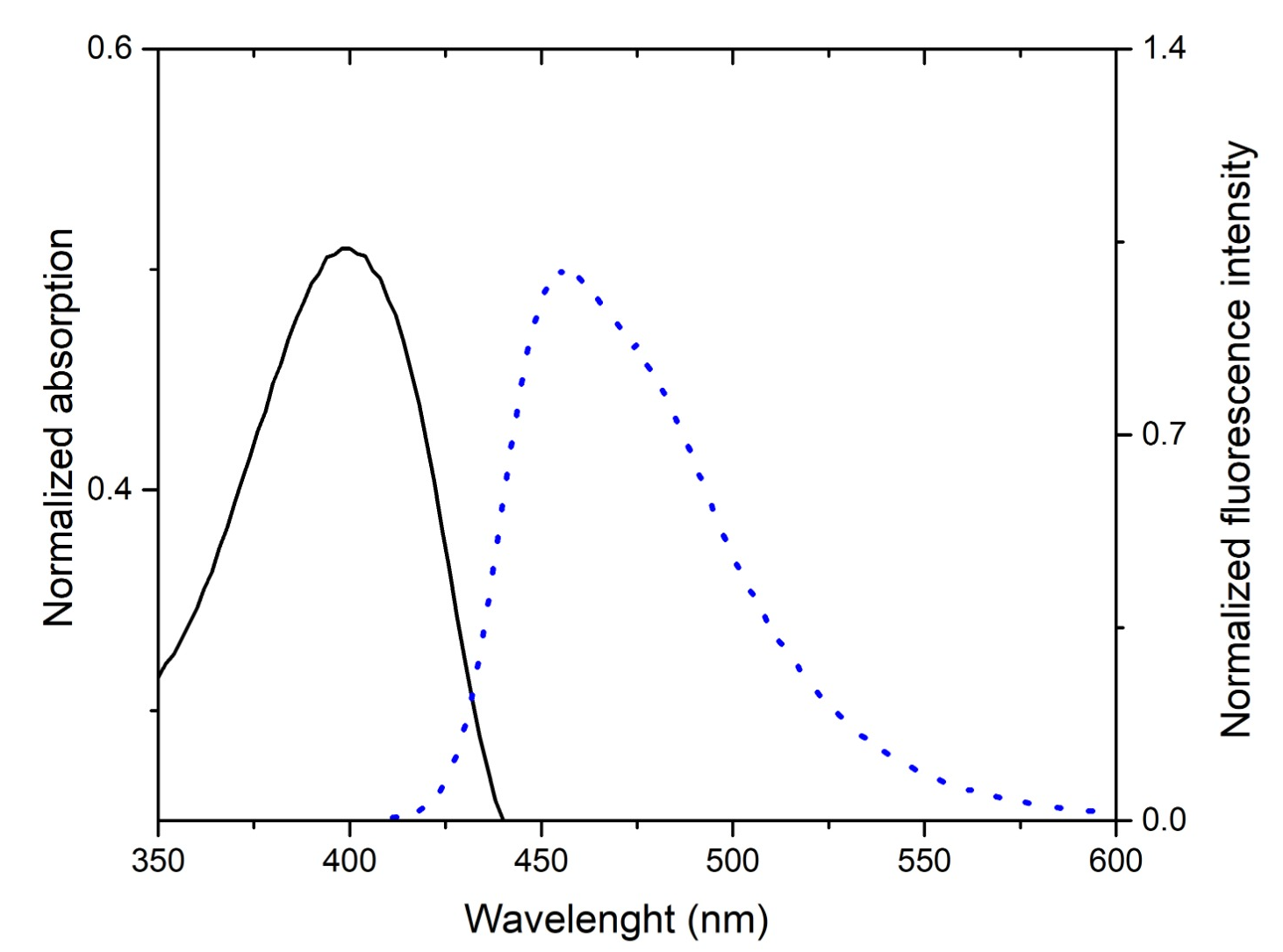


**Figure S21.** UV-Vis and Fluorescence Spectra of Compound **4a** in THF


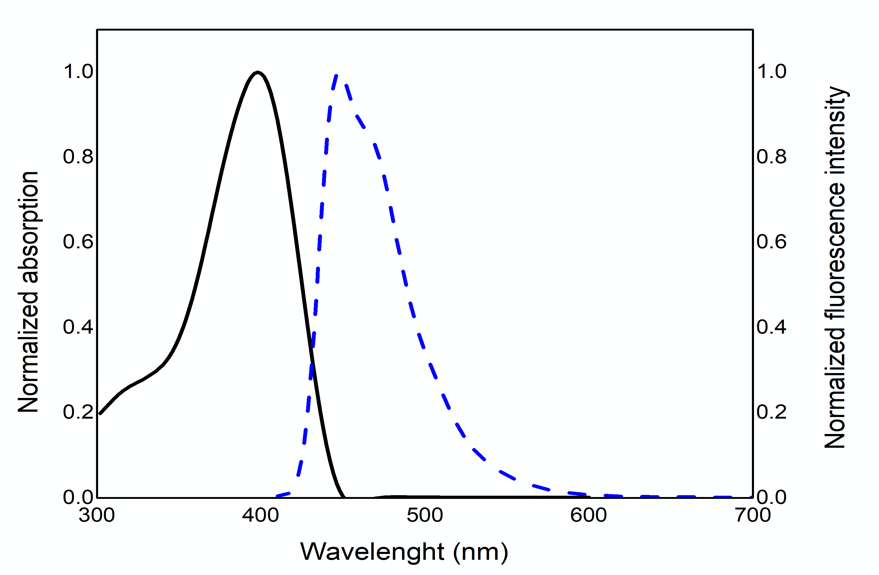


**Figure S22.** UV-Vis and Fluorescence Spectra of Compound **4b** in Toluene


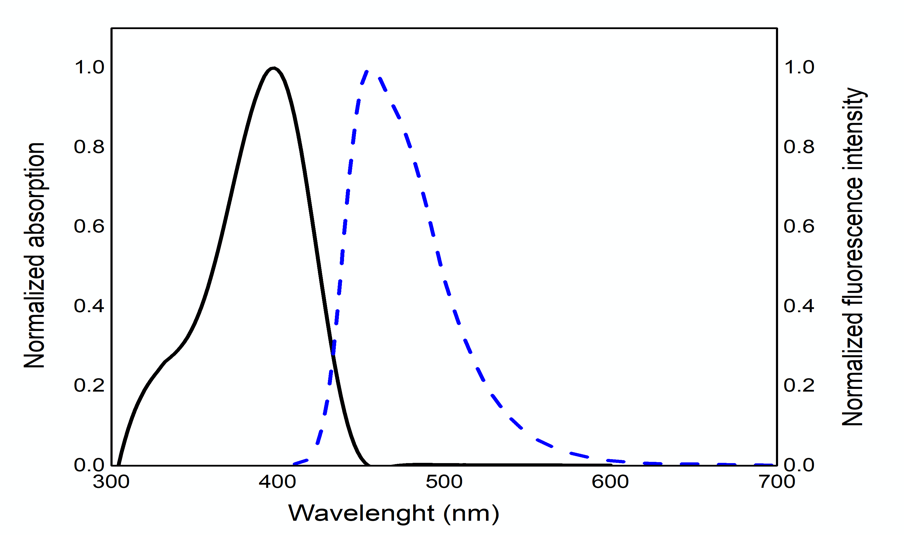


**Figure S23.** UV-Vis and Fluorescence Spectra of Compound **4b** in THF


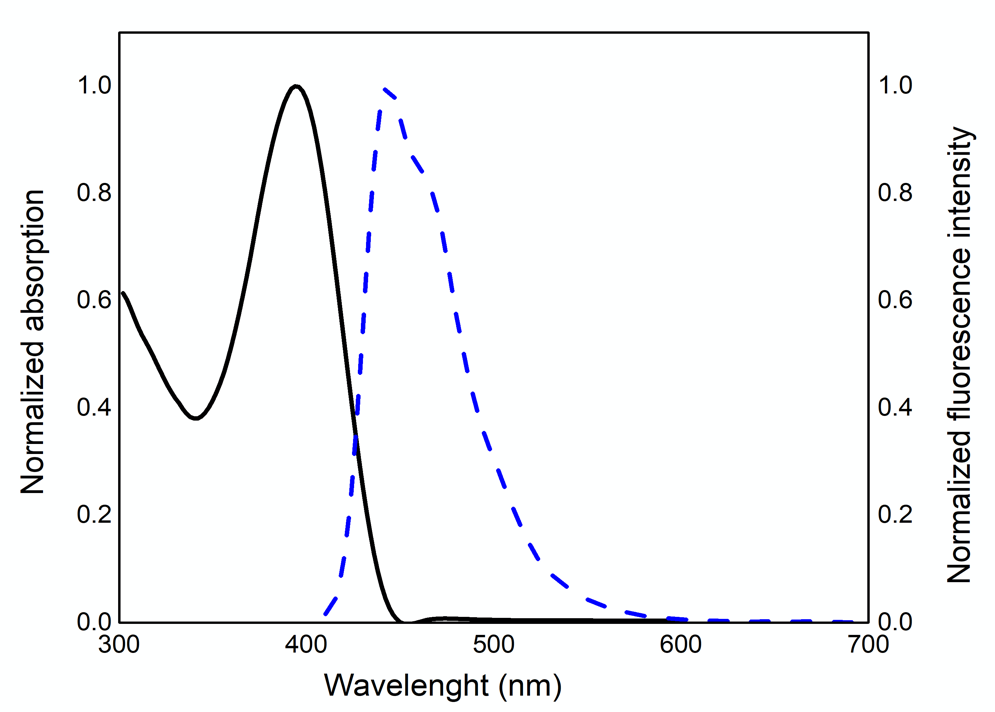


**Figure S24.** UV-Vis and Fluorescence Spectra of Compound **4c** in Toluene


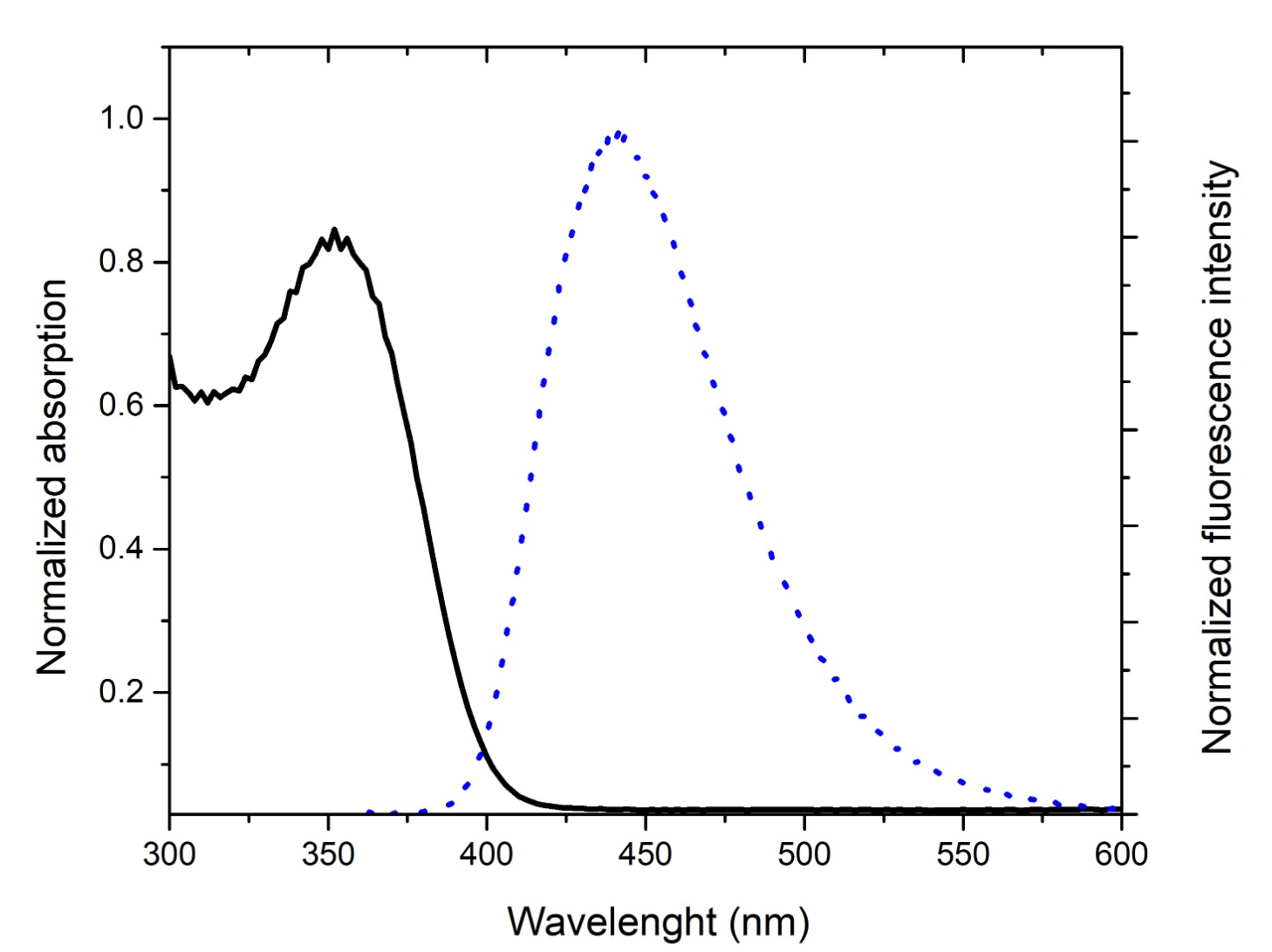


**Figure S25.** UV-Vis and Fluorescence Spectra of Compound **5a** in THF


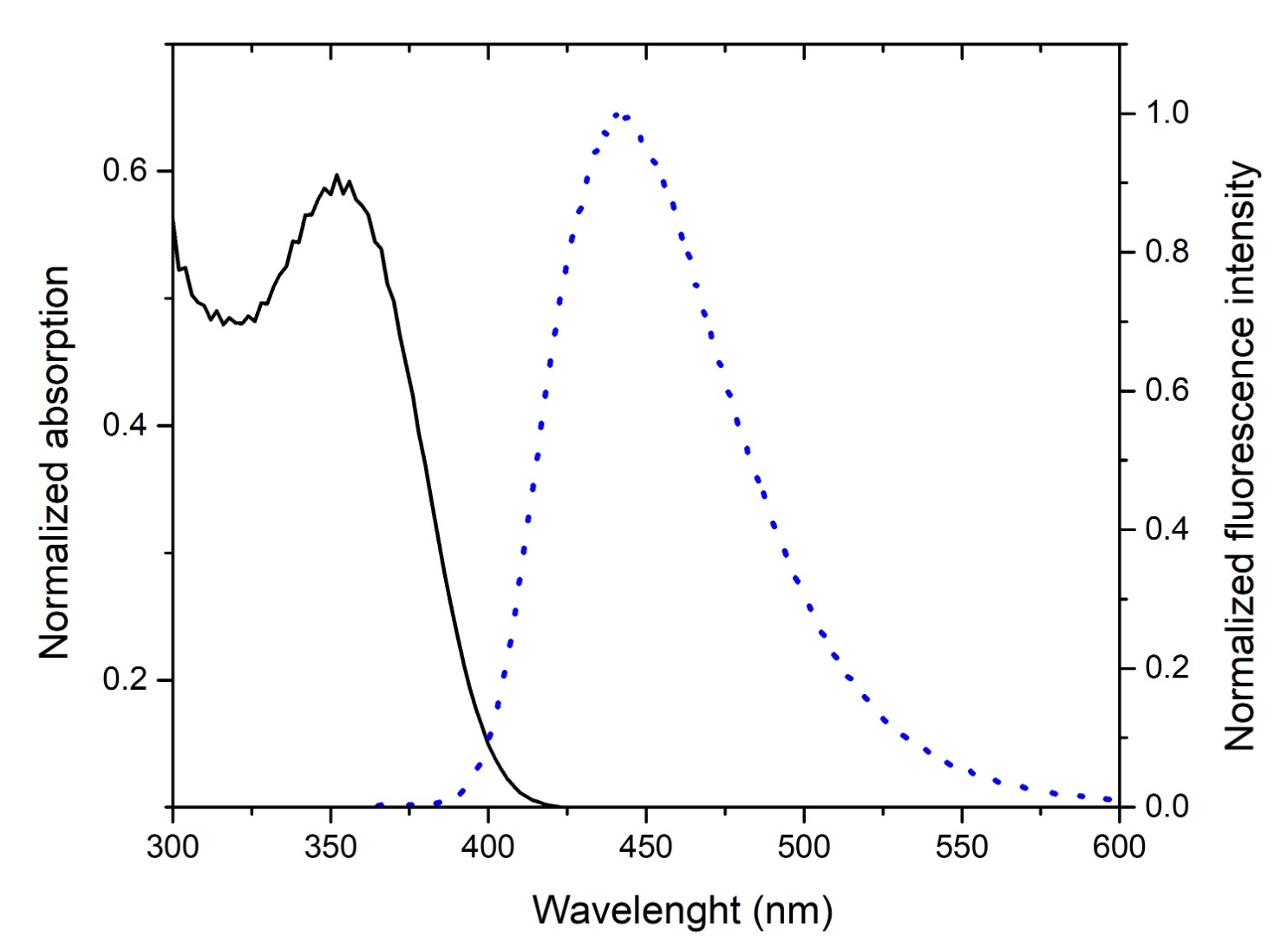


**Figure S26.** UV-Vis and Fluorescence Spectra of Compound **5a** in DCM


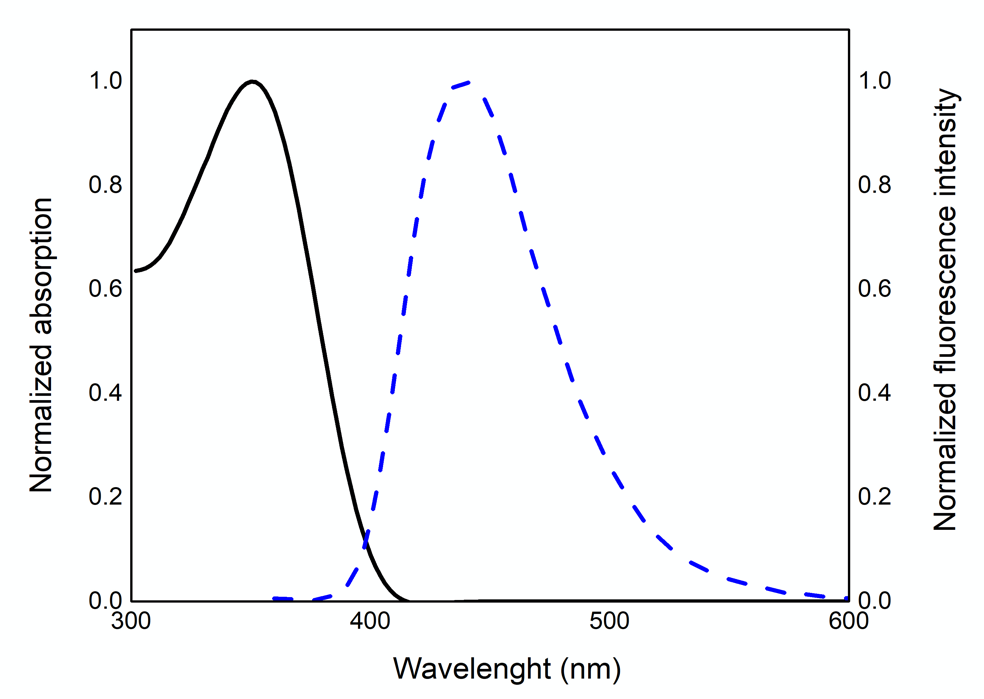


**Figure S27.** UV-Vis and Fluorescence Spectra of Compound **5b** in THF


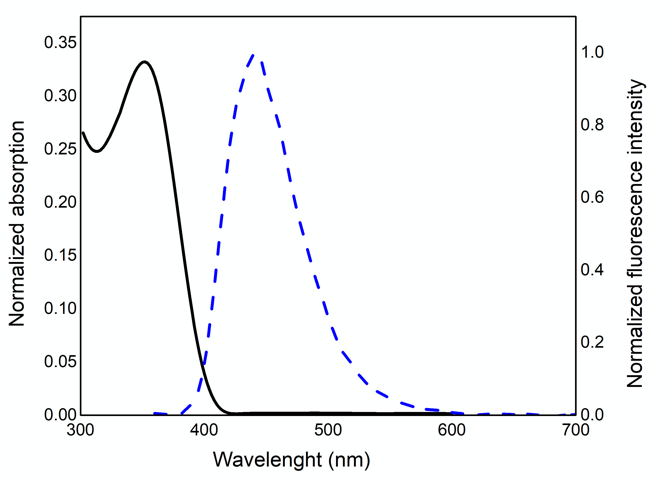


**Figure S28.** UV-Vis and Fluorescence Spectra of Compound **5b** in DCM
